# New insights into the responder/nonresponder divide in rectal cancer: Damage-induced Type I IFNs dictate treatment efficacy and can be targeted to enhance radiotherapy

**DOI:** 10.1038/s41419-023-05999-3

**Published:** 2023-07-26

**Authors:** Taylor P. Uccello, Maggie L. Lesch, Sarah A. Kintzel, Lauren B. Gradzewicz, Lillia Lamrous, Shawn P. Murphy, Fergal J. Fleming, Bradley N. Mills, Joseph D. Murphy, Angela Hughson, Gary Hannon, Jesse Garrett-Larsen, Haoming Qiu, Michael G. Drage, Jian Ye, Nicholas W. Gavras, David C. Keeley, Tanzy M. T. Love, Elizabeth A. Repasky, Edith M. Lord, David C. Linehan, Scott A. Gerber

**Affiliations:** 1 Department of Microbiology and Immunology, University of Rochester Medical Center, Rochester, NY USA; 2 Department of Surgery, University of Rochester Medical Center, Rochester, NY USA; 3 Department of Biomedical Engineering, University of Rochester, Rochester, NY USA; 4 Department of Obstetrics and Gynecology, University of Rochester Medical Center, Rochester, NY USA; 5 Department of Radiation Oncology, University of Rochester Medical Center, Rochester, NY USA; 6 Department of Pathology and Laboratory Medicine, University of Rochester Medical Center, Rochester, NY USA; 7 Department of Biostatistics and Computational Biology, University of Rochester Medical Center, Rochester, NY USA; 8 Roswell Park Comprehensive Cancer Institute, University at Buffalo, Buffalo, NY USA

**Keywords:** Rectal cancer, Immune cell death

## Abstract

Rectal cancer ranks as the second leading cause of cancer-related deaths. Neoadjuvant therapy for rectal cancer patients often results in individuals that respond well to therapy and those that respond poorly, requiring life-altering excision surgery. It is inadequately understood what dictates this responder/nonresponder divide. Our major aim is to identify what factors in the tumor microenvironment drive a fraction of rectal cancer patients to respond to radiotherapy. We also sought to distinguish potential biomarkers that would indicate a positive response to therapy and design combinatorial therapeutics to enhance radiotherapy efficacy. To address this, we developed an orthotopic murine model of rectal cancer treated with short course radiotherapy that recapitulates the bimodal response observed in the clinic. We utilized a robust combination of transcriptomics and protein analysis to identify differences between responding and nonresponding tumors. Our mouse model recapitulates human disease in which a fraction of tumors respond to radiotherapy (responders) while the majority are nonresponsive. We determined that responding tumors had increased damage-induced cell death, and a unique immune-activation signature associated with tumor-associated macrophages, cancer-associated fibroblasts, and CD8^+^ T cells. This signature was dependent on radiation-induced increases of Type I Interferons (IFNs). We investigated a therapeutic approach targeting the cGAS/STING pathway and demonstrated improved response rate following radiotherapy. These results suggest that modulating the Type I IFN pathway has the potential to improve radiation therapy efficacy in RC.

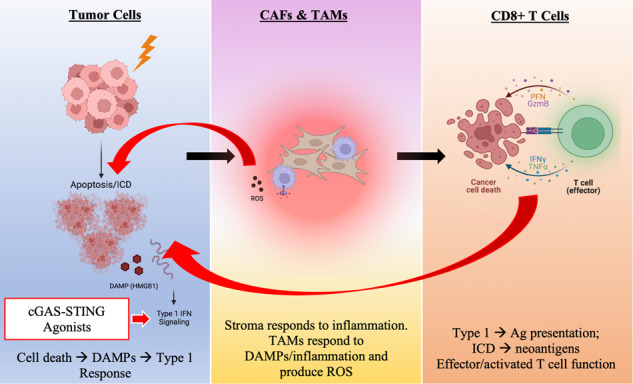

## Introduction

Colorectal cancer (CRC) is a devastating malignancy ranking as the third most common cancer diagnosis (10%) and second leading cause of cancer-related death in the United States [1]. Historically, treatment for patients diagnosed with early-stage rectal cancer (RC) was total mesorectal excision surgery (TMES) [2]. This surgery is particularly invasive as it requires partial removal of the rectum to eliminate the tumor, and some patients are rendered dependent on colostomy bags that significantly impact quality of life [3]. As a result, physicians and scientists are exploring additional therapeutic combinations to treat patients with RC.

Neoadjuvant therapy reduces the risk for local recurrence following surgical excision [4]. The current standard of care for RC is neoadjuvant short course radiotherapy (SCRT) or chemoradiotherapy (CRT) with the potential of some patients having to undergo excision surgery [5]. Fortunately, a fraction of patients initially respond to preoperative therapy (e.g. tumor regression) making TMES unnecessary for this cohort. Consequently, an approach known as Watchful Waiting (WW) has emerged following preoperative RT or CRT to postpone surgery, thus sparing rectal function, and offers a promising alternative to surgical excision for preserving quality of life [6]. Unfortunately, only 30% of patients demonstrate a clinical complete response (cCR) following preoperative therapy and are eligible for WW [7].

Numerous studies have attempted to identify why some patients respond to RT or CRT whereas many patients show no signs of tumor control. Unfortunately, the driving factor(s) that dictate this divide have not yet been identified. Furthermore, there are currently no biomarkers that indicate whether patients will respond. Therefore, we focused on modeling the responder/nonresponder phenomenon in a preclinical model of RC to translate our findings to the clinic.

We developed an orthotopic murine model of RC [8] that demonstrates a clinically relevant, responder/nonresponder phenotype when treated with SCRT (5 Gy/fraction, five fractions). A thorough analysis of the tumor microenvironment (TME) via RNA sequencing, immunohistochemistry (IHC), depletion studies, and preclinical therapeutic modulation identified Type I Interferon (IFN) signaling as a key pathway involved in mediating the response to RT. This result was dependent on cell death and damage-associated molecular pattern (DAMP) signaling and can be improved therapeutically using a small molecule agonist. These findings offer a promising clinical strategy to improve the responsiveness to RT for RC patients.

## Materials and methods

### Cell culture and growth kinetics

Murine Colon-38 (C57BL/6; American Type Culture Collection, Manassas Virginia, USA) and CT26 (BALB/c; Cellomics Technology, Halethorpe MD, USA) maintained in MAT/P (US Patent number 4.816.401) were supplemented with 2% Fetal Bovine Serum (FBS; GIBCO Thermo Fisher, Waltham MA, USA), 1% penicillin/streptomycin (Thermo Fisher), engineered to express luciferase (luc), and individual clones were selected by serial dilution. Growth kinetics were assayed by plating 1 × 10^5^ cells/well in 2 milliliter (mL) media and counted by trypan blue exclusion/hemocytometer at various timepoints.

### Murine model, SCRT, and BLI

All experiments were approved by the University Committee on Animal Resources, performed in compliance with the National Institute of Health (NIH) and University approved guidelines. Six- to eight-week-old age matched female C57BL/6J, BALB/c, or IFN-ɣ knock-out (KO) mice (Jackson Laboratory, Bar Harbor ME, USA) were subjected to a twelve-hour light/dark cycle and kept in individually ventilated cages. 2.5 × 10^4^ MC38-luc or CT26-luc tumor cells were implanted orthotopically [8] and irradiated on days 9–13 with 5 Gray (Gy) RT per fraction for 5 consecutive fractions using a Small Animal Radiation Research Platform (SARRP) [8] with tumor burden measured by In vivo Imaging system (IVIS; Perkin Elmer, Waltham MA, USA).

For the ectopic model, 1 × 10^5^ MC38-luc tumor cells injected intramuscularly (i.m.) into the left flank muscle of C57BL/6 mice were locally irradiated from days 9–13 (5 Gy per fraction for five fractions). Mice were confined in a plastic case with only the tumor-bearing flank positioned directly on the uncovered section of the irradiator and tumor size was monitored.

### Definition of response

Tumors were targeted for radiotherapy using titanium fiducial markers surgically implanted on either side of the rectal tumor on day 8 (one day prior to the start of SCRT). Response was determined based on percent change in tumor BLI on day 13 (after the final dose of radiotherapy) compared to baseline BLI (calculated on day 6) where responders had a stable or negative percent change and nonresponders demonstrated increased tumor burden.

### RNA sequencing preparation and analysis

Tumors were harvested on day 14 following SCRT, weighed, designated as responders or nonresponders, and prepared for bulk sequencing [9]. Tumor-associated macrophages (TAMs; CD45+, CD11b+, Ly6C−, Ly6G−, F480+), CD8^+^ T cells (CD45+, CD8b+), MC38-GFP tumor cells (CD45− GFP+) and cancer-associated fibroblasts (CAFs; CD45^−^ CD31^−^ GFP^−^ PDGFRα^+^ Podoplanin^+^ Ly6C^+^) were sequenced.

For TAMs, samples were processed by filtering out genes with low expression and performing a variance stabilizing transformation resulting in filtered genes assessed by Principal Component Analysis (PCA) with principal component (PC)-1 demonstrating a 65% variance and PC2 a 14% variance. The Euclidian distance was calculated based on the expression vectors between the untreated average to each irradiated sample and plotted against tumor size. The accension numbers for the RNA sequencing data reported in this paper are GSE211991 and GSE227738. Analyses code available upon request.

### Flow cytometry

Tumors were extracted, weighed, and homogenized in collagenase (Sigma Aldrich, St. Louis MO, USA) diluted in Hanks Balanced Salt solution (HBSS; Sigma Aldrich), followed by further homogenization in the gentle Macs homogenizer (Miltenyl Biotec, Bergisch Gladbach). Single cell suspensions were filtered (70-micron), stained with antibody cocktail in PAB (phosphate buffered saline (PBS), 0.1% sodium azide, 1% bovine serum albumin; Millipore Sigma, Burlington MA, USA) for 30 min, and fixed in CytoPerm/CytoFix (BD Bioscience, San Jose CA, USA) for 20 min followed by analysis on LSRII and FlowJo software (version 10.8.1).

### Measuring phosphorylated gamma H2A.x

Mice injected orthotopically with MC38-luc tumor cells received a single dose of 5 Gy targeted to the orthotopic tumor and were sacrificed three hours following RT. Tumors were processed for flow cytometry using fluorescently conjugated anti-CD45 and anti-phosphorylated gamma H2A Histone Family Member X (H2A.x) antibodies.

### Monitoring hypoxia in the TME

MC38-luc orthotopic mice were injected retro-orbitally with 150 micrograms (µg) of a 2-nitroimidazole based hypoxia marker (EF5; Millipore Sigma) or PBS three hours prior to sacrifice. Tumors were harvested and stained for flow cytometry using an ELKCy3 antibody (75 ug/ml) [8].

### Histology

Excised tumors fixed in 10% neutral buffered formalin (Azer Scientific, Morgantown PA, USA) were paraffin embedded, sectioned into 5- micron slices, and stained with anti-cleaved caspase 3 (Cell Signaling, Danvers MA, USA), anti-High Mobility Group Box I (HMGB1; Abcam, Cambridge UK), or anti-Calreticulin (Abcam), followed by secondary antibody (Vector Laboratories, Newark CA, USA) and counter stained with hematoxylin. Marker expression was determined by Aperio Image Scope algorithm.

### Spatial transcriptomics

MC38-luc tumors harvested on day 11 (midway through SCRT) where 5-micron histological sections were adhered to spatially barcoded capture areas of a Visium Gene Expression Slide (10X Genomics, Pleasanton CA, USA). Following tissue placement, slide drying, and overnight incubation in a desiccator, the slide was deparaffinized, hematoxylin and eosin (H&E) stained, and imaged using a VS120 Slide Scanner (Olympus, Tokyo Japan) at 20X magnification. Sequence ready libraries were constructed (10X Genomics, CG000407) with final libraries sequenced on the NovaSeq 6000 sequencer (Illumina, Shanghai China) to obtain >50,000 reads per spot and analyzed using the Seurat package in R (Uniform Manifold Approximation and Projection (UMAP) analysis, RNA assessment, bubble plots), and Loupe Browser (gene overlay).

### IFNα and IFNβ ELISA

MC38-luc tumor-bearing mice treated with either three doses of 5 Gy or the full SCRT dose were sacrificed on days eleven and fourteen, respectively. Tumors were harvested, weighed, snap frozen in Lysis II buffer, thawed, and manually homogenized. Homogenates were centrifuged at 300 × G for five minutes and supernatants analyzed using the Mouse IFN Beta High Sensitivity ELISA kit and Mouse IFN Alpha All Subtypes High Sensitivity ELISA kit (Pbl Assay Science, Piscataway NJ, USA). Concentrations normalized to grams of tumor tissue.

### CD8^+^ T cell depletion

CD8 + T cells were depleted from MC38-luc orthotopic tumor-bearing mice by injecting 200 µg/100 microliter (µL) PBS anti-mouse CD8α (BioxCell, Lebanon NH, USA) or IgG control s.c. every third day from days 5–19 and monitored by IVIS (Perkin Elmer).

### IFNAR neutralizing antibody treatment

The IFN alpha receptor (IFNAR) was blocked in MC38-luc orthotopic tumor-bearing mice by injecting 200 µg/100 µL PBS anti-mouse IFNAR (BioxCell) s.c. every third day from days 9–18 and monitored by IVIS.

### cGAMP treatment

MC38-luc orthotopic tumor-bearing mice were injected with 10 µg/mouse 2’3’-Cyclic Guanosine Monophosphate-Adenosine Monophosphate (cGAMP; Millipore Sigma) intratumorally on day eight. Mice received SCRT, and immediately following each dose of RT were intravenously injected in the tail vein with 20 µg cGAMP (in PBS). Tumor burden was monitored by IVIS or mice sacrificed on day twenty-six and primary tumors weighed.

### Human CRC survival curve stratified by STING1

Human patients with any stage of CRC from the Human Protein Atlas were stratified based on high or low expression of stimulator of interferon genes (STING1) and survival was plotted. Survival plot terminated at 6 years. Ethical Approval and Consent for Use: The supplementary data used in this manuscript is from the publicly available Human Protein Atlas database and does not require ethics approval and consent for use.

### Statistical analysis

Statistical analysis performed in GraphPad Prism 8 Software. We performed an a priori power calculation was performed using G*Power software with a stated power of 0.9, α of 0.05 and a determined effect size of 0.729 to calculate appropriate n. BLI growth curves plotted as the geometric mean with standard deviation (SD) and survival determined by the Mantel-Cox test (*p* < 0.05). All other data presented as mean +/− SD. Significance for single comparisons determined by unpaired nonparametric Mann-Whitney T test and multiple group comparisons assessed by ordinary one-way Analysis of Variance (ANOVA) with multiple comparison post hoc tests.

## Results

### Radiotherapy elicits a responder/nonresponder phenotype

**Fig. 1:**
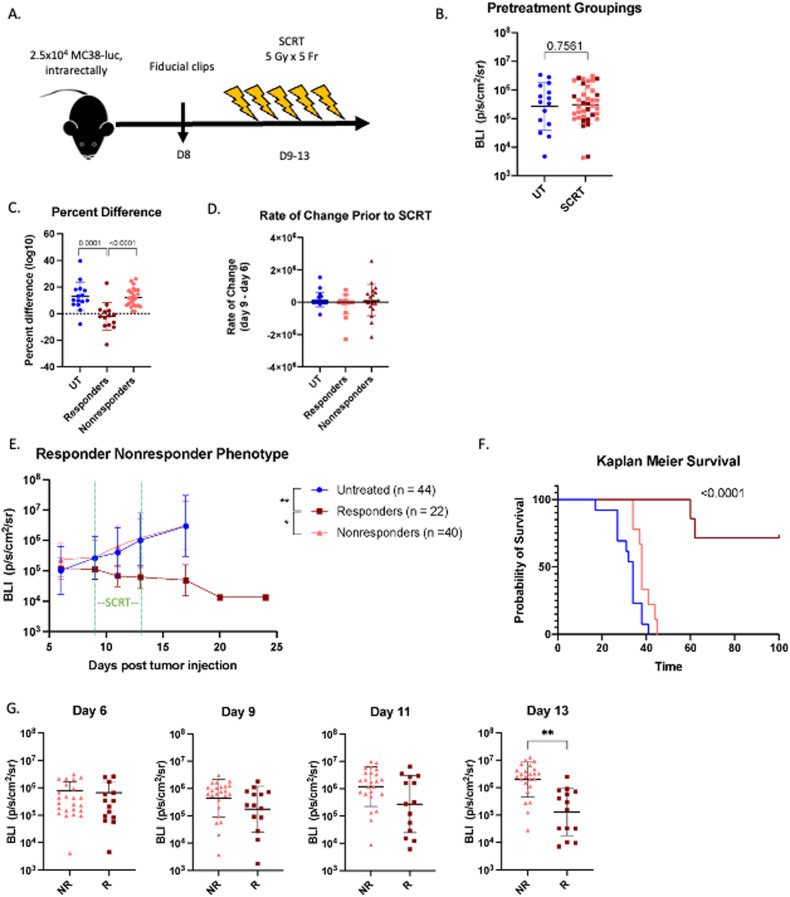
SCRT elicits a responder/nonresponder phenotype. **A** Mice injected intrarectally with 2.5 × 10^4^ MC38-luc cells on day 0. Titanium fiducial clips incorporated on either side of the tumor at day 8, and SCRT administered on days 9–13. **B** Mice were prearranged to ensure equivalent geometric means before treatment (maroon = responders, pink = nonresponders, blue = untreated; UT). **C** Fold change in tumor size from days 9–13. **D** Rate of growth from days 6 to 9 (prior to SCRT). **E** Tumor burden determined by IVIS over 24 days; (n) refers to number of animals per group. Green dotted line indicates SCRT treatment. **F** Overall survival (Kaplan Meier Survival Curve). **G** Tumor BLI retrospectively grouped as responders (R) and nonresponders (NR) at day 6 (before SCRT), day 9 (1 fraction), day 11 (3 fractions), day 13 (final fraction). **B**, **G** – Statistical significance by *t*-test. **C**, **D**, **E** – Statistical significance by ANOVA (**E** - on d17). **F** – Statistical significance by log rank Mantel-Cox test.

RC patients treated with preoperative RT demonstrate a heterogeneous response; whereas a subset of patients’ tumors initially respond to RT (20%), most patients are nonresponsive [10]. To study this divide we utilized our established orthotopic model of RC and clinically relevant, targeted SCRT [8]. Mice were injected with luciferase-expressing MC38 tumor cells intrarectally, titanium fiducial clips were surgically inserted on opposing sides of the tumor on day eight, and tumors were targeted with SCRT from days nine through thirteen (five Gy per dose in five consecutive fractions) (Fig. 1A). Mice were randomly grouped such that each group had tumors with equal geometric means (Fig. 1B). The responder/ nonresponder designation was determined by percent difference in tumor burden (BLI) as measured from the start of SCRT (day nine) to the end (day thirteen). Responders had a stable or negative percent change as opposed to nonresponders who demonstrated increased tumor burden (Fig. 1C) despite all animals having equal tumor growth prior to SCRT (Fig. 1D). A fraction of mice treated with SCRT (37%) respond to therapy and have reduced tumor burden, whereas the majority of mice, although treated with an identical dose of SCRT, demonstrate no reduction in tumor growth (Fig. 1E). Additionally, mice with radioresponsive tumors exhibited significantly enhanced overall survival compared to non-radioresponsive tumors (Fig. 1F).

Mice were retrospectively grouped as responders or nonresponders, and tumor sizes at individual timepoints during SCRT were analyzed to pinpoint when the difference in tumor burden emerged (Fig. 1G). Tumor burden was equal across all groups prior to the start of treatment on day six and remained consistent after the first fraction of RT (day nine). However, by the third day of RT (day eleven), differences emerged, although it was not until the final dose of SCRT (day thirteen) that the responding tumors were significantly smaller than the nonresponding. This demonstrated that the divide in response is not predetermined, but develops during the course of SCRT.

We demonstrated tissue targeting is precise in all animals and is likely not the cause of the varied response. γH2A.x is rapidly and transiently phosphorylated in response to double stranded DNA breaks (DSB). Phosphorylated (p)-ɣH2A.x was significantly upregulated in all tumor tissue harvested three hours following a single dose of targeted SCRT compared to unirradiated controls and compared to adjacent tissue in irradiated individuals (Supplementary Fig. 1a–d). To further rule out that the magnitude of response is not dictated by RT targeting, MC38-luc cells were injected i.m. into the flank and the entire leg was irradiated with an identical 5 Gy by five fractions SCRT schedule (Supplementary Fig. 2a). Although less clinically relevant, the advantage of this model is that the entire leg muscle was irradiated, making it unlikely the tumor would be outside the field of treatment. In this scenario, the leg tumors also demonstrated a responder/nonresponder curve following SCRT (Supplementary Fig. 2b).

Intratumor hypoxia may alter effectiveness of RT as oxygen is essential for the generation of reactive oxygen species (ROS) that promote DSB [11]. Although SCRT decreased the percentage of EF5^+^ (hypoxic) immune (CD45^+^) and nonimmune (CD45^−^) populations, there are no significant differences when stratified based on responders/nonresponders (Supplementary Fig. 3). These data suggest that SCRT can reduce intratumoral hypoxia, but it is not preferentially reduced in responding tumors.

To investigate whether tumor cell clonality of the heterogenous MC38-luc cell line was contributing to the divided SCRT response, we generated three MC38-luc clones that were characterized in vitro (Supplementary Fig. 4) and subsequently implanted orthotopically where each clone also exhibited both responders and nonresponders to SCRT (Supplementary Fig. 5). To further generalize beyond MC38, we characterized clones of CT26-luc in vitro (Supplementary Fig. 6a–c) and selected one clone to test orthotopically in BALB/c mice. SCRT treated CT26-luc tumor-bearing mice similarly exhibited a responder/nonresponder divide (Supplementary Fig. 6d–f).

Collectively, our orthotopic responder/nonresponder model recapitulates the divide seen in patients following treatment. We next assessed what factors dictate this clinically-relevant response.

### Although the quantity of immune infiltration does not differ, the phenotype of TAMs from responding tumors exhibit distinct polarization

The efficacy of RT is largely mediated by the immune system [12]. We hypothesized that the magnitude of response to SCRT is driven by either the quantity or quality of immune infiltrate. We focused on day fourteen as this was the earliest timepoint after SCRT where differences in tumor weight were identified (Supplementary Fig. 7a). There were no differences in the ratio of immune to nonimmune cells, nor in the number of specific immune subtypes aside from inflammatory monocytes (IMs) (Supplementary Fig. 7b, c). Therefore, we performed a more in-depth analysis of the phenotype of infiltrating immune cells by bulk RNA sequencing of sorted immune populations. TAMs from the tumor homogenate of untreated, responding, and nonresponding tumors were selected and sequenced (Supplementary Fig. 8a) as they were the most abundant myeloid population in the TME (Supplementary Fig. 7c).

**Fig. 2:**
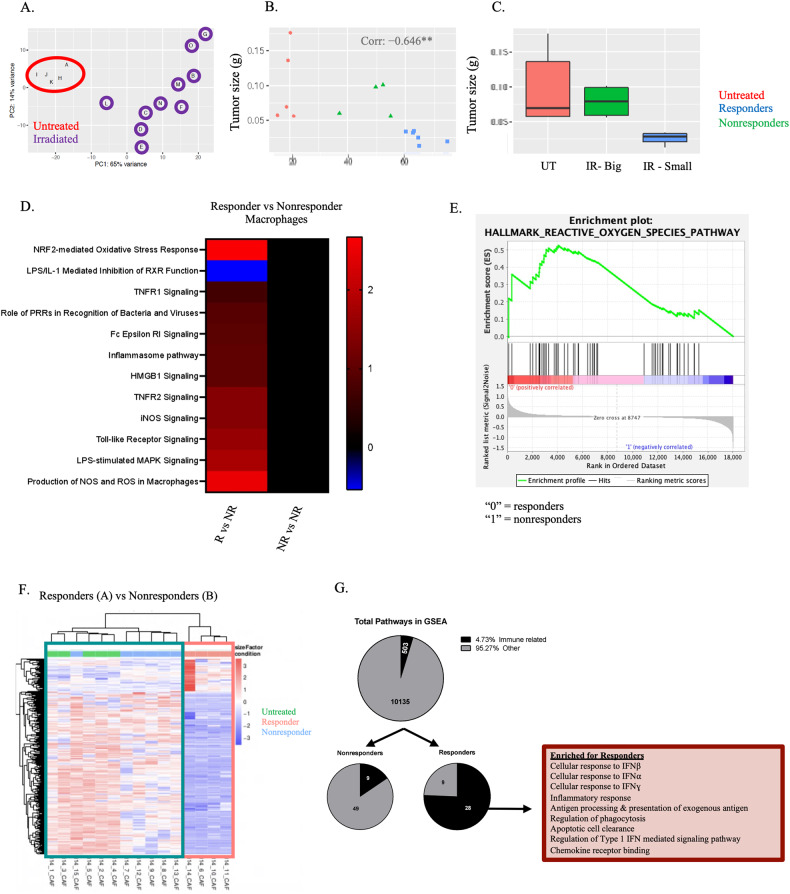
RNA sequencing performed on TAMs and CAFs demonstrate distinct inflammatory phenotypes in responding tumors. **A** PCA plot of bulk RNA sequencing from TAMs isolated from d14 untreated or irradiated (SCRT; d9-13) tumors. Red circle = untreated; UT. Purple circles = irradiated. **B** The Euclidian distance between the untreated average and each irradiated sample plotted against tumor size. Red = untreated, blue = responders, green = nonresponders and quantified in (**C**). **D** DAMP/PAMP pathway analysis of differentially expressed genes (DEGs) from responder or nonresponder TAMs compared to TAMs from untreated tumors. **E** DEGs from responding sorted TAMs compared to nonresponding TAMs analyzed by gene set enrichment analysis (GSEA). **F** DEGs between responding and nonresponding cancer-associated fibroblasts (CAFs) plotted on a hierarchical clustering heatmap. Teal box = nonresponders and untreated and pink box = responders. **G** GSEA analysis identified differences in predominately immune related signatures in CAFs from responder tumors. *N* = 5 untreated, 10 irradiated (6 responder, 4 nonresponders for TAMs; 4 responders, 6 nonresponders for CAFs).

RNA sequencing results for the TAMs were plotted on a PCA plot. Untreated samples formed a tight cluster; however, the irradiated samples were dispersed throughout the plot (Fig. 2A). The Euclidian distance between each individual irradiated sample and the center of the untreated cluster were calculated. Values were correlated to tumor size resulting in a significant negative correlation (Fig. 2B, refer to Experimental Methods and Materials). The four irradiated TAM samples that were farthest away from the untreated average (and most genetically distinct) were also the four smallest tumors (e.g., responders) (Fig. 2C). In contrast, the samples that were most similar to the untreated in terms of genetic profile were the largest tumors (e.g., nonresponders). This served as independent validation of our responder/nonresponder classification system. These results suggest that TAMs from nonresponding samples are genetically similar to untreated while TAMs from responding samples have a more distinct genetic profile.

We performed pathway analysis on differentially expressed genes (DEGs) between responding and nonresponding TAMS and determined that responding TAMs were enriched for DAMP (HMGB1) and toll like receptor (TLR) signaling (Fig. 2D). Furthermore, we determined by gene set enrichment analyses (GSEA) that TAMs from responding tumors were enriched in ROS production (Fig. 2E). These data suggest TAMs from responding tumors exhibit a heightened response to damage.

### Heightened levels of inflammation in TME of responding tumors influences CAFs

In addition to immune infiltration, the TME consists of other stromal cells. We sorted and sequenced CAFs from tumors ex vivo to determine whether the transcriptome of CAFs differed between responding or nonresponding tumors. Activated CAFs were classified as CD45^−^ CD31^−^ GFP^−^ PDGFRα^+^ podoplanin^+^ Ly6C^+^ (Supplementary Fig. 9a). Distinct genetic signatures were observed when DEGs between responding and nonresponding CAFs were assessed by hierarchical clustering (Fig. 2F). Our CAF dataset was compared to the 10,538 gene sets compiled by the BROAD Institute and analyzed by GSEA (Fig. 2G). Of the fifty-eight significantly enriched pathways for the nonresponders, only nine of those pathways were related to immune regulation and function (16%) (Fig. 2G). However, of the thirty-seven significantly enriched pathways for the responders, twenty-eight were related to the immune system (76%), including the response to both Type I and Type II IFNs. These data demonstrate that, similar to TAMs, intratumoral CAFs from responding tumors exhibit a unique inflammatory signature.

### MC38-luc cells purified from responding tumors display distinct phenotypes associated with damage and cell death

**Fig. 3:**
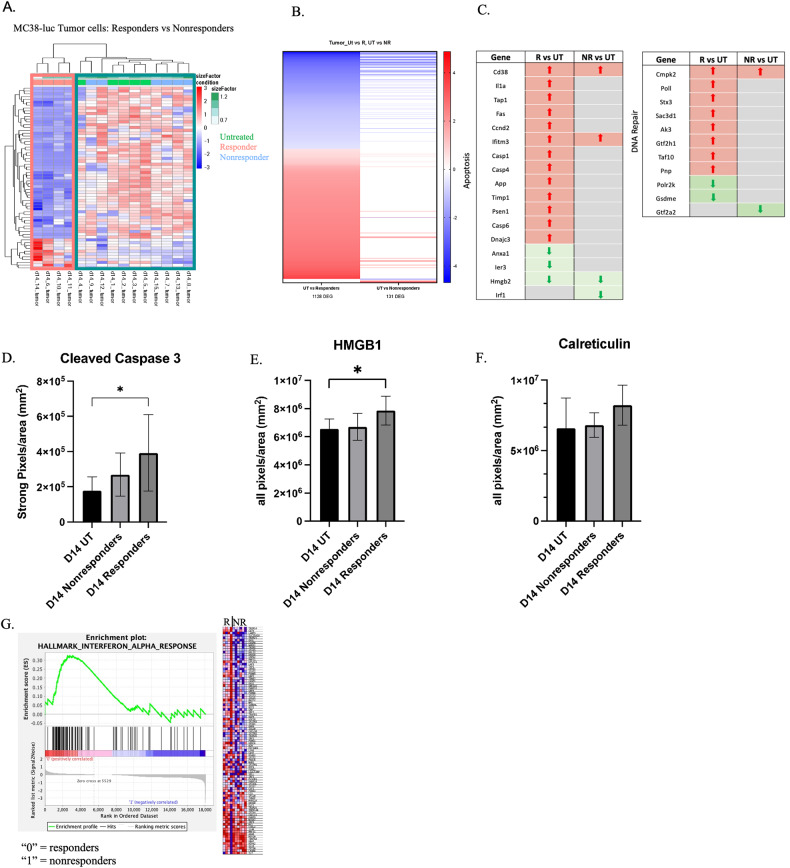
MC38 cells from responding tumors have increased cell death and damage. **A** DEGs between responder and nonresponder tumor cells plotted on a differential clustering heatmap. Teal box = nonresponders and untreated, pink box = responders. **B** Quantification of DEGs comparing untreated vs. responders to untreated vs, nonresponders. **C** DEGs from responding and nonresponding tumors compared to published gene sets for apoptosis/DNA damage. Red = upregulated genes; green = downregulated genes; both are compared to untreated. *N* = 5 untreated, 10 irradiated (4 responders, 6 nonresponders). **D** Immunohistochemistry performed on d14 untreated or irradiated tumors against anti- cleaved caspase 3, HMGB1 (**E**), calreticulin (**F**). *N* = 10 untreated, 7 nonresponders, 4 responders. **G** Pathway analysis of DEGs from responding (R) sorted tumor cells compared to nonresponding (NR). **D**, **E**, **F** - Statistical significance by ANOVA.

We next investigated whether the transcriptome of RT responsive or nonresponsive *tumor* cells differed. MC38-GFP tumor cells were flow sorted from tumors ex vivo on day fourteen and RNA sequencing was performed (Supplementary Fig. 10a). Nonresponding tumor cells were similar to untreated tumor cells in terms of DEGs whereas the responding samples had a distinct genetic profile (Fig. 3A). Furthermore, the magnitude of response was heightened in responding tumors, which exhibited almost 10-times more DEGs when compared to nonresponding tumors (1128 vs 131 DEGS when compared to untreated tumors, respectively: Fig. 3B).

We compared MC38-GFP sequencing results to a publicly available gene list for apoptosis and determined that cell death-associated genes were upregulated in responding tumor cells, but not in nonresponding tumor cells (Fig. 3C). Additionally, genes involved in DNA repair were specifically enhanced in responding samples (Fig. 3C). We validated our RNA-sequencing results by performing IHC on day fourteen tumor sections for markers of cell death (cleaved caspase-3) and DAMP release (HMGB1 or calreticulin). Image analysis indicated that responding samples had significantly increased levels of cleaved caspase-3 and HMGB1, and a trending increase in calreticulin (Fig. 3D–F). Furthermore, increasing the amount of cell death by elevating RT doses (*e.g*., 8 Gy/fraction & 12 Gy/fraction) resulted in more responders (e.g., 60% & 80% respectively). (Supplementary Fig. 11).

Lastly, GSEA Pathway Analysis directly comparing responding tumor cells against nonresponding resulted in IFNα response as the only significantly enriched hit (Fig. 3G). Type I IFNs are made in response to viral infection, or, more relevant in our model, in response to cell death and damage. Therefore, combined with the increases in cleaved caspase 3, HMGB1, and calreticulin determined by IHC, enrichment in Type I IFNs in the responding samples indicated elevated levels of damage and immunogenic cell death (ICD) compared to the nonresponding tumors.

### Type I IFNs are increased in responding tumor regions compared to nonresponding and untreated tumors

**Fig. 4:**
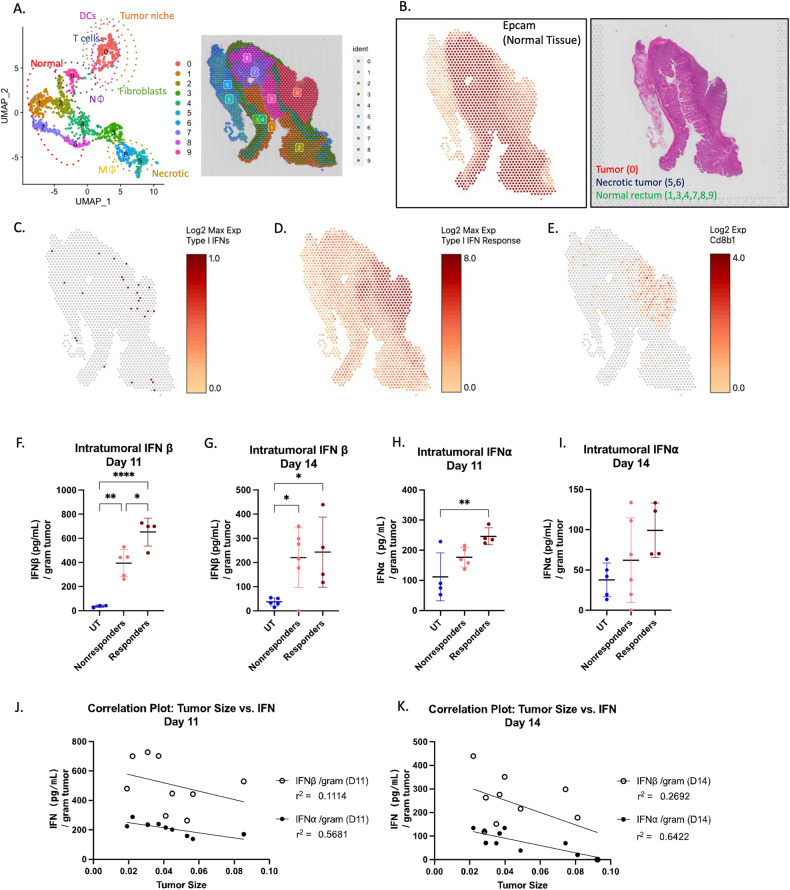
Type I IFNs are increased early in responding tumors compared to nonresponding and untreated tumors. **A** Uniform Manifold Approximation and Projection analysis of irradiated tumor section with clusters based on cell type. Refer to Supplementary Fig. 12 for group classification. **B** Tissue section of EpCAM expression and corresponding H&E. **C** Tissue section demonstrating expression of Type I IFN, (**D**) Type I IFN response genes (refer to Table 1), (**E**) CD8β1 gene. Intratumoral IFNβ concentration of d11 (**F**) and d14 (**G**) or IFNα on d11 (**H**) of d14 (**I**) as determined by ELISA. **J** Correlation plots between tumor size and IFN concentration on day 11 or (**K**) 14. *N* = 4–5 untreated, 5–6 nonresponders, and 4 responders for ELISA. **F**, **G**, **H**, **I** – Statistical significance by ANOVA.

To confirm that Type I IFNs were elevated primarily in irradiated tumor tissue, and not in adjacent non-malignant regions, we performed spatial transcriptomics on irradiated tumor sections and classified the different tissue regions based on UMAP clustering (Fig. 4A, Supplementary Fig. 12a, b). We identified regions of normal tissue (regions 1–4, 7–9) based on epithelial cellular adhesion molecule (*EpCAM*) expression and malignant regions based on histological assessment along with a lack of EpCAM expression and positive cytokeratin (*Cdk6*) staining (region 0; Fig. 4B, Supplementary Fig. 12b). Expression of Type I IFN mRNAs were predominantly localized to the tumor region (Fig. 4C). To complement this finding, we assessed various genes associated with responsiveness to Type I IFNs (Table 1) and illustrated that the inflammatory response to Type I IFNs were similarly colocalized only within the tumor region (Fig. 4D). We referenced the transcriptomes of the cells in this region and determined that aside from tumor cells, the major immune cells in this section were dendritic cells (DCs; *Itgax/ Cd11c*) (Supplementary Fig. 12b) and CD8^+^ T cells (Fig. 4E), both of which are immune populations influenced by Type I IFNs [13].

**Table 1:** A list of included genes in the “response to Type I IFN” dataset used for spatial transcriptomics.

Gene Symbol	Gene Name
Ifit1	Interferon-induced protein with tetratricopeptide repeats 1
Ifit2	Interferon-induced protein with tetratricopeptide repeats 2
Rsad2	Radical S-adenosyl methionine domain containing 2
Dxd58	DEAD/H box helicase 58
Ifih1	Interferon induced with helicase C domain 1
Ifitm1	Interferon induced transmembrane protein 1
Oas1	2’-5’ oligoadenylate synthetase 1 A
Usp18	Ubiquitin specific peptidase 18

We quantified the intratumoral concentration of Type I IFN protein in responding and nonresponding tumors by ELISA. Intratumoral IFNβ protein concentrations were significantly increased in responding tumors compared to nonresponding and untreated tumors on day eleven (Fig. 4F). These values decreased by 50% on day fourteen, however, irradiated tumors still demonstrated a significant increase compared to untreated (Fig. 4G). Likewise, intratumoral IFNα concentrations in the responders demonstrated significant increases when compared to untreated tumors on day 11 (Fig. 4H) and were still trending higher at day 14 (Fig. 4I). The amount of Type I IFNs negatively correlated with tumor size on days eleven and fourteen, where smaller tumors (i.e., responders) had increased IFNα/β concentration compared to larger tumors (i.e., nonresponders) (Fig. 4J, K). These results confirm the importance of Type I IFNs, especially *early on*, in promoting a response to SCRT.

### CD8 + T cells are essential for driving a response and are heavily influenced by Type I IFNs

**Fig. 5:**
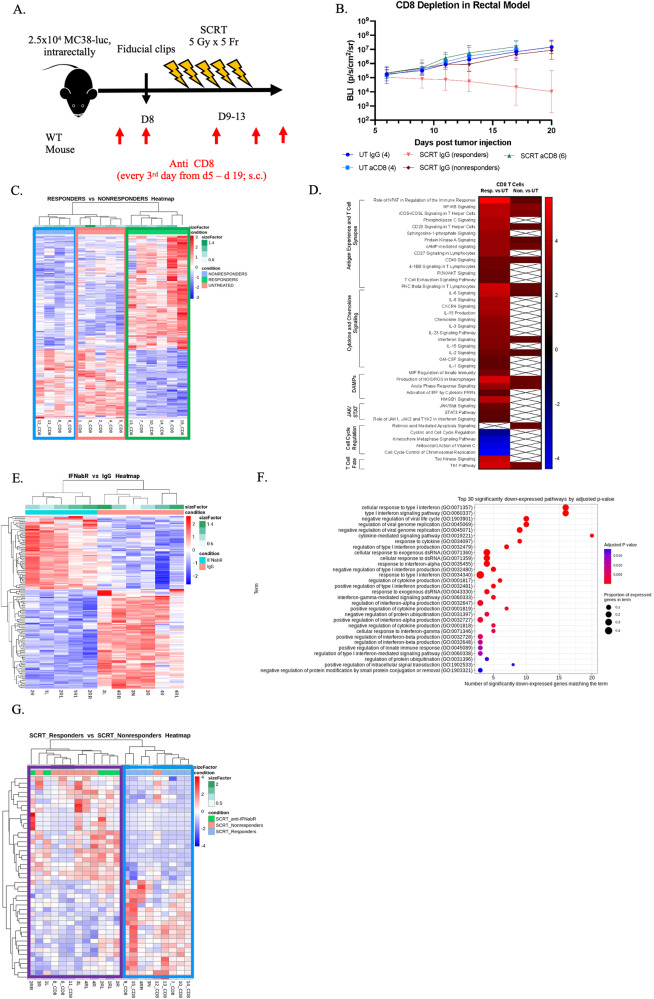
CD8^+^ T cells are essential, phenotypically distinct in responding vs. nonresponding tumors, and require Type I IFN signaling. **A** CD8^+^ T cells were depleted from tumor-bearing mice prior to SCRT treatment. **B** Tumor burden monitored by IVIS for 20 days. **C** DEGs between responding CD8^+^ T cells and nonresponding CD8^+^ T cells plotted on a hierarchical clustering heatmap. Blue box = nonresponders, green = responders, and pink = untreated. **D** Pathway heatmap of DEGs. *N* = 5 untreated and 10 irradiated (4 nonresponders, 6 responders) for sequencing wildtype CD8^+^ T cells. **E** CD8^+^ T cells from d14 irradiated tumors of mice treated with IFNAR blocking antibodies or IgG control. *N* = 6 IgG and 5 IFNAR treated. **F** Top 30 significantly downregulated pathways of CD8^+^ T cell from IFNAR-treated mice compared to IgG-treated mice. **G** Hierarchical clustering heatmap comparing CD8^+^ T cells from SCRT responders (blue box) from WT mice to SCRT nonresponders from WT mice including IFNAR treated CD8^+^ T cells (purple box).

Figure 4 identified abundant CD8^+^ T cells in the tumor region that colocalized with Type I IFNs. Depletion experiments determined that CD8^+^ T cells were essential for generating a response to SCRT (Fig. 5A, B). Based on these data, we hypothesized that responder tumors maintained higher quality CD8^+^ T cells compared to nonresponders. To test this, we sorted CD8^+^ T cells from irradiated or untreated tumors and performed bulk RNA sequencing (Supplementary Fig. 13). Unbiased hierarchical clustering of DEGs grouped CD8^+^ T cells from nonresponding tumors with those in untreated tumors, suggesting these two groups were similar to each other. In contrast, CD8^+^ T cells from responder tumors displayed clear differences in gene expression from both nonresponders and untreated CD8^+^ T cells (Fig. 5C). Pathway analysis revealed only minor differences between the nonresponding and untreated CD8^+^ T cells, however striking differences were observed between responder and untreated CD8^+^ T cells (Fig. 5D). These results included pathways related to antigen experience, cytokine and chemokine signaling, response to DAMPs, cell cycle regulation, and fate. Collectively, these results indicated a more activated T cell transcriptome in responding tumors.

To explore the impact of Type I IFN signaling on T cell effector status, we treated tumor-bearing mice with an anti-IFNAR neutralizing antibody followed by SCRT and harvested CD8^+^ T cells for bulk RNA sequencing. The transcriptome of CD8^+^ T cells treated with IgG exhibited significant differences from the T cells harvested from the IFNAR-treated mice (Fig. 5E). The top 30 pathways that were significantly downregulated when Type I IFN signaling was blocked by the IFNAR antibody are hallmarks of the Type I response, including regulation and signaling of the Type I IFN pathway, viral response genes, and cytokine production (Fig. 5F). Furthermore, CD8^+^ T cells from IFNAR treated mice grouped more closely with nonresponders while responding CD8^+^ T cells formed their own distinct cluster (Fig. 5G). These data suggest that heightened Type I IFN signaling from responding tumors promotes an activated/effector-like T cell phenotype.

### Type I IFN drives the responder tumor phenotype following SCRT

**Fig. 6:**
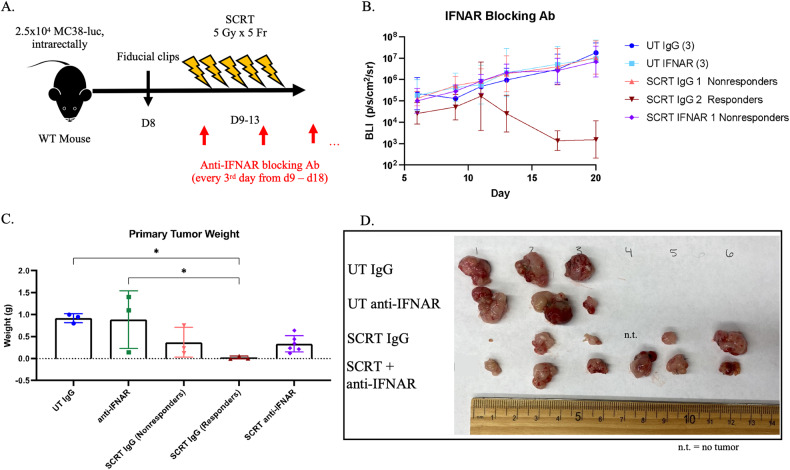
Type I IFN signaling is necessary for the response to SCRT. **A** MC38-luc tumor-bearing mice treated subcutaneously with anti-IFNAR every 3 days from day 9 through 18+/− SCRT. **B** Tumor burden monitored by IVIS. **C** Mice sacrificed on d20 and tumor burden assessed by weight and (**D**) gross visualization. n.t. = no tumor. *N* = 3–6. Representative data from two separate experiments. **C** – Statistical significance by ANOVA.

Figure 5 demonstrated that blockade of Type I IFN signaling renders CD8^+^ T cells similar to nonresponders/untreated cells. We postulated this blockade will result in a poor response to SCRT and predominately nonresponder tumors. Neutralization of Type I IFN signaling with an IFNAR blocking antibody (Fig. 6A) resulted in 100% of IFNAR-treated mice being nonresponsive to SCRT (Fig. 6B) with larger tumor burden upon sacrifice on day twenty compared to the IgG-treated controls (Fig. 6C, D). Our results highlight the importance of Type I IFN in dictating SCRT efficacy.

### Activating the cGAS-STING pathway increased the number of responders

There are various mechanisms by which Type I IFN can be triggered in response to damage. For example, extracellular DNA is recognized by over ten different cytosolic receptors, one of which is cGAS, which upon binding cytoplasmic DNA becomes catalytically active and generates cGAMP. cGAMP binds STING and causes translocation to the golgi resulting in Type I IFN secretion [14]. Using a human CRC dataset collated by the Human Protein Atlas we determined that patients with high STING expression had improved overall survival compared to patients with low STING expression (Supplementary Fig. 14) justifying a therapeutic intervention to target this pathway.

**Fig. 7:**
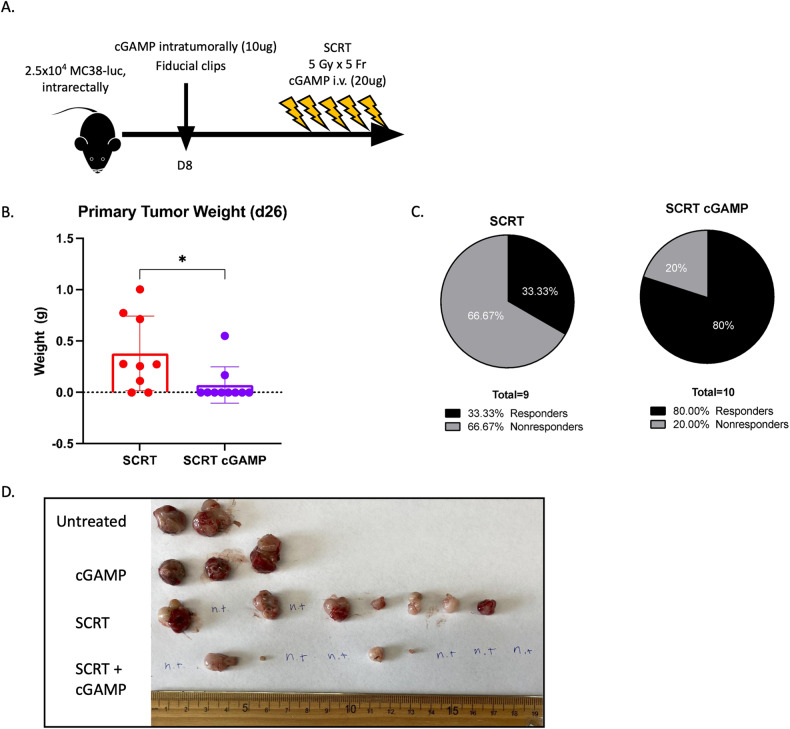
cGAMP therapy improves SCRT efficacy. **A** MC38-luc tumor-bearing mice treated with intratumoral cGAMP on day 8 followed by SCRT and intravenous cGAMP injections from days 9–13. **B** Mice sacrificed on d26 and tumor burden assessed by weight. **C** The percentage of responders vs. nonresponders calculated for SCRT treatment alone and SCRT + cGAMP combination therapy. **D** Primary tumors visualized by gross examination on day 26. n.t. = no tumor. *N* = 2–10. Representative data from two separate experiments. **B** – Statistical significance by *t*-test.

Tumor-bearing mice were treated with cGAMP intratumorally on day eight followed by intravenous administration daily throughout SCRT (Fig. 7A), and mice sacrificed on day twenty-seven. SCRT-treated tumors demonstrated the typical 30–40% response rate, however mice treated with SCRT in combination with cGAMP demonstrated an 80% response rate where eight of the ten individuals had minor, if any, residual primary tumor (Fig. 7B, C, D). These results demonstrate that cGAMP and SCRT combinatorial therapy significantly improve localized response compared to SCRT alone.

## Discussion

The data presented demonstrate that SCRT induces a responder/nonresponder phenotype in multiple murine models of RC, indicative of the response observed clinically. Our findings suggest that ICD drives acute Type I IFN signaling resulting in a more activated subset of CD8^+^ T cells and TAMs in the responding samples compared to the nonresponding samples. Although not clinically relevant, increasing the dose to 12 Gy/fraction results in an 80% response rate, compared to the 40% response rate when 5 Gy is administered. This experiment informed us that it was possible to adjust the ratio of responders, and that radiation-induced cell death may be a contributing factor. Alternatively, and more clinically relevant, we determined that amplifying the cGAS/STING signaling pathway by therapeutically agonizing cGAS results in an increase in the percentage of responders to SCRT.

We demonstrated that SCRT efficacy was significantly blunted when animals were treated with a blocking antibody against Type I IFN signaling. Type I IFNs encompass a large family of structurally related monomeric pleiotropic cytokines ^18^ where IFNα and IFNβ are the most well-characterized [15]. In addition to cellular damage [16], viral or bacterial invasion, extracellular self-nucleic acid (tumor-secreted DNA)-induced cGAS/STING signaling, or binding of other non-nucleic acids (DAMPs) via TLRs [14] all drive Type I IFN production. Type I IFNs activate several immune subsets, including T cells, via the IFNAR to stimulate immunity following RT-induced cellular damage. For example, Type I IFNs increase cytotoxicity and cytokine production of CD8^+^ T cells [17] and promote durable T cell memory responses following viral infection [18]. These studies extend beyond responses to viruses as Type I IFNs have recently been accredited with priming an immune response against tumor cells, especially in the context of RT [19]. Many of these effects are mediated by triggering of cGAMP/STING. This is further emphasized by data demonstrating that heightened STING expression in human CRC correlated with increased overall survival (Supplementary Fig. 14).

A key question is why is there increased ICD in a subset of tumors even through all received similar RT doses? We ruled out differences in oxygenation status, and the clonality experiment confirmed that genetic predispositions of individual tumor cells were not a driver of response rate. Further genetic analysis of the tumors by RNA sequencing determined that the MC38 tumor cells harvested ex vivo from irradiated tumors are undergoing DNA damage and apoptosis, however this may be a readout of RT efficacy, rather than an indicator of RT-sensitivity. Further experimentation to determine what causes increased tumor cell death in a subset of tumors is needed to entirely elucidate this phenotype, however it is possible that epigenetic differences emerge when tumor cells are implanted and irradiated in vivo.

In accordance with the robust literature surrounding Type I IFN signaling, Type I IFNs were approved by the Food and Drug Administration (FDA) for cancer treatment in 1986. However, to our knowledge, no current clinical trials explore the combination of IFN treatment alongside SCRT for RC. Based on the data presented here, we propose that quantification of intratumoral Type I IFN protein concentrations and DAMP secretion of irradiated RC biopsies could serve as a predictive biomarker to determine who is likely to respond preoperatively. Additionally, our data supports a non-randomized Phase II clinical trial for patients with locally advanced, Stage II/III RC. The goal of this trial would be to demonstrate that cGAMP in combination with the standard of care treatment results in decreased tumor size and invasion at the time of resection, or alternatively that combination treatment results in a higher rate of responders who can delay TMES, compared to patients treated with CRT alone.

## Supplementary information

confirmation of additional author

reproducibility checklist

Supplemental Figure 1

Supplemental Figure 2

Supplemental Figure 3

Supplemental Figure 4

Supplemental Figure 5

Supplemental Figure 6

Supplemental Figure 7

Supplemental Figure 8

Supplemental Figure 9

Supplemental Figure 10

Supplemental Figure 11

Supplemental Figure 12

Supplemental Figure 13

Supplemental Figure 14

Supplemental Figure Legends
