# Supplementary material for: New insights into the responder/nonresponder divide in rectal cancer: Damage-induced Type I IFNs dictate treatment efficacy and can be targeted to enhance radiotherapy
Source: Cell Death Dis. 2023 Jul 26;14(7):470. doi: 10.1038/s41419-023-05999-3 (PMC10372053; doi:10.1038/s41419-023-05999-3)
Supplement: Supplementary file 1 — confirmation of additional author [file 41419_2023_5999_MOESM1_ESM.pdf]

From: Gerber, Scott <Scott_Gerber@URMC.Rochester.edu>
Sent: Wednesday, June 28, 2023 4:46 PM
To: Lesch, Maggie <Maggie_Lesch@URMC.Rochester.edu>; Gradzewicz, Lauren <lgradzew@u.Rochester.edu>; Uccello, Taylor <TUCCELLO@mgh.harvard.edu>; Murphy, Joseph D. <Joseph_Murphy@URMC.Rochester.edu>; Linehan, David <David_Linehan@URMC.Rochester.edu>; Qiu, Haoming (Carl) <Haoming_Qiu@URMC.Rochester.edu>; Murphy, Shawn P. <Shawn_Murphy@urmc.rochester.edu>; Fleming, Fergal <Fergal_Fleming@URMC.Rochester.edu>; Mills, Bradley <Bradley_Mills@URMC.Rochester.edu>; Hughson, Angie <Angie_Hughson@urmc.rochester.edu>; Jesse Garrett-Larsen <jgarrettlarsen97@gmail.com>; mdrage.pathology@gmail.com <mdrage.pathology@gmail.com>; Repasky Subjeck, Elizabeth <Elizabeth.Repasky@RoswellPark.org>; Nicholas Gavras <nicholasgavras2@gmail.com>; Love, Tanzy <Tanzy_Love@URMC.Rochester.edu>; Lord, Edith <Edith_Lord@urmc.rochester.edu>; lilialamrous9@gmail.com <lilialamrous9@gmail.com>; Cameron Keeley <dckeeley@gmail.com>; Ye, Jian <Jian_Ye@URMC.Rochester.edu>; Kintzel, Sarah <skintzel@u.Rochester.edu>
Subject: Approval need to Taylor's paper

Dear all,
After the last revision of Taylor's paper, we added Gary Hannon to the author list.

Per Cell Death and Disease's rules, all authors need to confirm this addition.

Could you just reply to this email stating "Confirm to addition of Gary Hannon". I will then send these on to finalize submission.

Thank you,

Scott

Scott A. Gerber Ph.D.
Associate Professor
Departments of Surgery, Microbiology/Immunology, & Radiation Oncology
Co-Director, Center for Tumor Immunology Research
Gerber Lab
University of Rochester Medical Center

Fleming, Fergal
Gerber, Scott
scott

Confirm to addition of Gary Hannon

thanks

fergal

Fergal Fleming, MD, MPH, FRCSI, FACS
Associate Professor of Surgery and Oncology,
Director, Surgical Health Outcomes and Research Enterprise (SHORE)
University of Rochester Medical Center

Nicholas Gavras <nicholasgavras2@gmail.com>
Gerber, Scott
Confirm to addition of Gary Hannon

Jesse Garrett-Larsen <jgarrettlarsen97@gmail.com>
Gerber, Scott
I confirm to addition of Gary Hannon.

Jesse

Mills, Bradley
Gerber, Scott
Confirm to addition of Gary Hannon.

Bradley N. Mills, PhD
Research Assistant Professor
Department of Surgery
Director, Biobank Shared Resource
Wilmot Cancer Institute, RM 3-0771E
University of Rochester Medical Center
601 Elmwood Avenue
Box SURG
Rochester, NY 14642

Office: (585) 275-7272
Cell: (585) 813-3445

Ye, Jian
Gerber, Scott
I confirm to addition of Gary Hannon to the author list.
Jian
Sent from my iPhone

Qiu, Haoming (Carl)
Gerber, Scott
I agree thanks

Haoming (Carl) Qiu MD
Associate Professor
Radiation Oncology
University of Rochester Medical Center

Repasky Subjeck, Elizabeth <Elizabeth.Repasky@RoswellPark.org>
Gerber, Scott;
Lesch, Maggie;
Gradzewicz, Lauren;
Uccello, Taylor <TUCCELLO@mgh.harvard.edu>;
Murphy, Joseph D.;
Linehan, David;
Qiu, Haoming (Carl);
Murphy, Shawn P.;
Fleming, Fergal;

Mills, Bradley;
Hughson, Angie;
Jesse Garrett-Larsen <jgarrettlarsen97@gmail.com>
I confirm the addition of Gary Hannon to the authorship of this paper,
Thank you,
Elizabeth Repasky

Murphy, Shawn P.
Gerber, Scott;
Lesch, Maggie;
Gradzewicz, Lauren;
Uccello, Taylor <TUCCELLO@mgh.harvard.edu>;
Murphy, Joseph D.;
Linehan, David;
Qiu, Haoming (Carl);
Fleming, Fergal;
Mills, Bradley;
Hughson, Angie;
Jesse Garrett-Larsen <jgarrettlarsen97@gmail.com>
"Confirm to addition of Gary Hannon"

Shawn P. Murphy PhD
He/Him/His
Associate Professor
Department of Obstetrics and Gynecology
University of Rochester School of Medicine
Box 668
601 Elmwood Avenue
Rochester, NY 14642
Tel: 585-273-3910

Lesch, Maggie
Gerber, Scott
I confirm to addition of Gary Hannon

Sarah Kintzel <skintzel@u.rochester.edu>
Gerber, Scott
Lesch, Maggie;
Gradzewicz, Lauren;
Uccello, Taylor <TUCCELLO@mgh.harvard.edu>;
Murphy, Joseph D.;
Linehan, David;
Qiu, Haoming (Carl);
Murphy, Shawn P.;
Fleming, Fergal;
Mills, Bradley;
Hughson, Angie;
Jesse Garrett-Larsen <jgarrettlarsen97@gmail.com>
"Confirm to addition of Gary Hannon"

Sarah A. Kintzel

University of Rochester
M.E. Engineering Management '24
B.S. Biomedical Engineering | Cell & Tissue Engineering
Research Scientist II | Curia Global
Sarah.Kintzel@curiaglobal.com

Love, Tanzy
Gerber, Scott

Confirm to addition of Gary Hannon

Tanzy

On Jun 28, 2023, at 15:46, Gerber, Scott <Scott_Gerber@urmc.rochester.edu> wrote:

Hughson, Angie
Gerber, Scott
Lesch, Maggie;
Gradzewicz, Lauren;
Uccello, Taylor <TUCCELLO@mgh.harvard.edu>;
Murphy, Joseph D.;
Linehan, David;
Qiu, Haoming (Carl);
Murphy, Shawn P.;
Fleming, Fergal;
Mills, Bradley;
Jesse Garrett-Larsen <jgarrettlarsen97@gmail.com>;
mdrage.pathology@gmail.com
Confirm the addition of Gary Hannon.

Thank you,
Angela Hughson

Linehan, David
Gerber, Scott
confirmed

Lauren Gradzewicz <lgradzew@u.rochester.edu>
Gerber, Scott
"Confirm to addition of Gary Hannon"

Best,
Lauren Gradzewicz

Uccello, Taylor <TUCCELLO@mgh.harvard.edu>
Gerber, Scott
Confirm the addition of Gary Hannon.

Thanks, Scott!

-Taylor Uccello

Lord, Edith
Gerber, Scott
Confirm to addition of Gary Hannon.

Edith Lord

Joe M <josephdmurphy93@gmail.com>
Gerber, Scott
Hi Scott,

Sorry for the late response to this.
I can't access my URMC email at this time.

Please take this as my approval for adding Gary Hannon as an author to this paper.

Let me know if you need anything else!

Joe

Lilia Lamrous <lilialamrous9@gmail.com>
Hughson, Angie
Gerber, Scott;
Lesch, Maggie;
Gradzewicz, Lauren;
Uccello, Taylor <TUCCELLO@mgh.harvard.edu>;
Murphy, Joseph D.;
Linehan, David;
Qiu, Haoming (Carl);
Murphy, Shawn P.;
Fleming, Fergal;
Mills, Bradley;
Jesse Garrett-Larsen <jgarrettlarsen97@gmail.com>
Confirm to addition of Gary Hannon.

Thank you.

Lilia LAMROUS

Michael Drage <mdrage.pathology@gmail.com>
Murphy, Shawn P.
Gerber, Scott;
Lesch, Maggie;
Gradzewicz, Lauren;
Uccello, Taylor <TUCCELLO@mgh.harvard.edu>;
Murphy, Joseph D.;
Linehan, David;
Qiu, Haoming (Carl);
Fleming, Fergal;

Mills, Bradley;
Hughson, Angie;
Jesse Garrett-Larsen <jgarrettlarsen97@gmail.com>

"Confirm to addition of Gary Hannon"

Thanks again for including me!
Best,
Mike
Sent from my iPhone
