# Supplementary figures and images for: New insights into the responder/nonresponder divide in rectal cancer: Damage-induced Type I IFNs dictate treatment efficacy and can be targeted to enhance radiotherapy
Source: Cell Death Dis. 2023 Jul 26;14(7):470. doi: 10.1038/s41419-023-05999-3 (PMC10372053; doi:10.1038/s41419-023-05999-3)

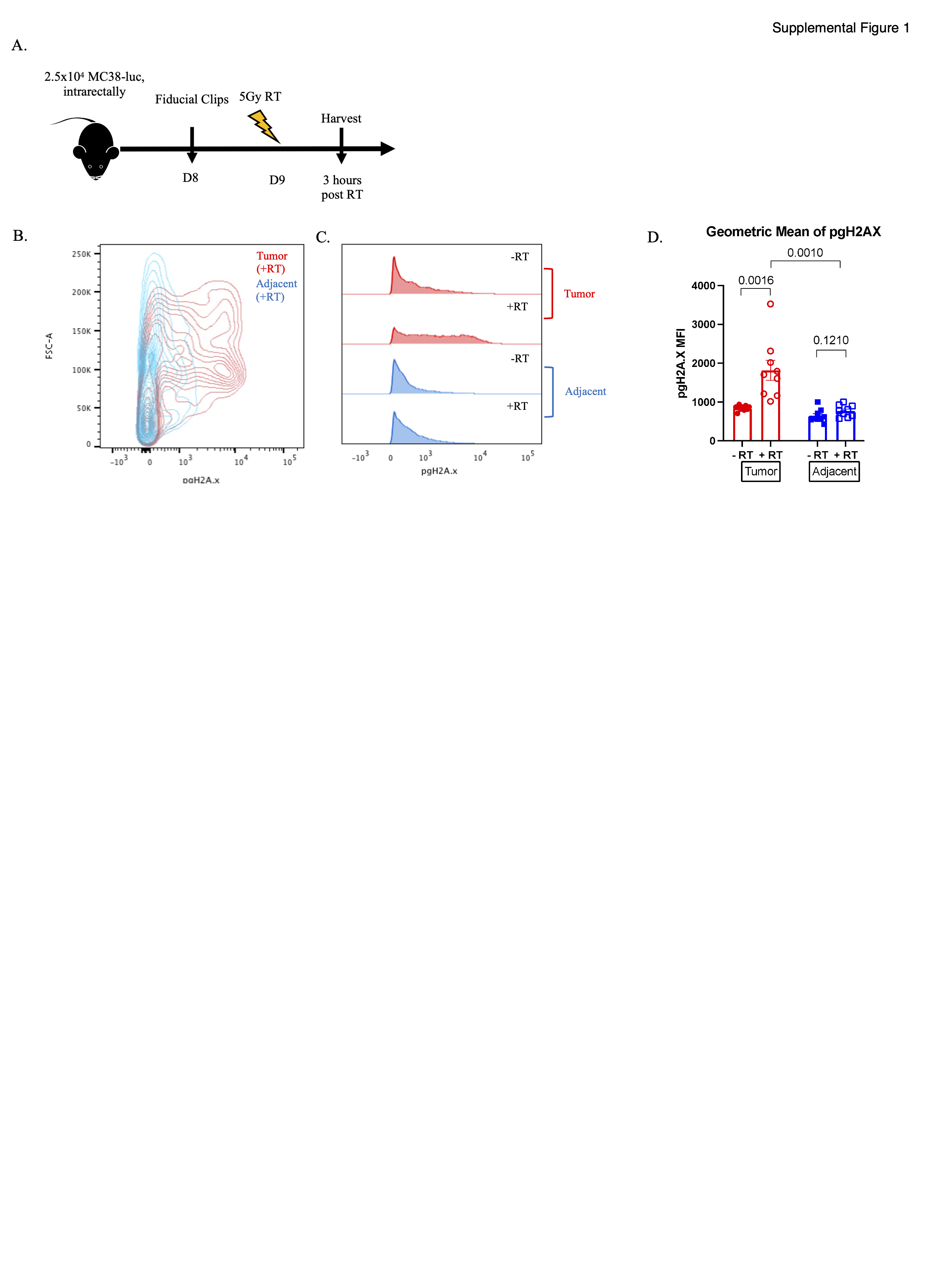

Supplement: Supplementary file 3 — Supplemental Figure 1 [file 41419_2023_5999_MOESM3_ESM.tif]

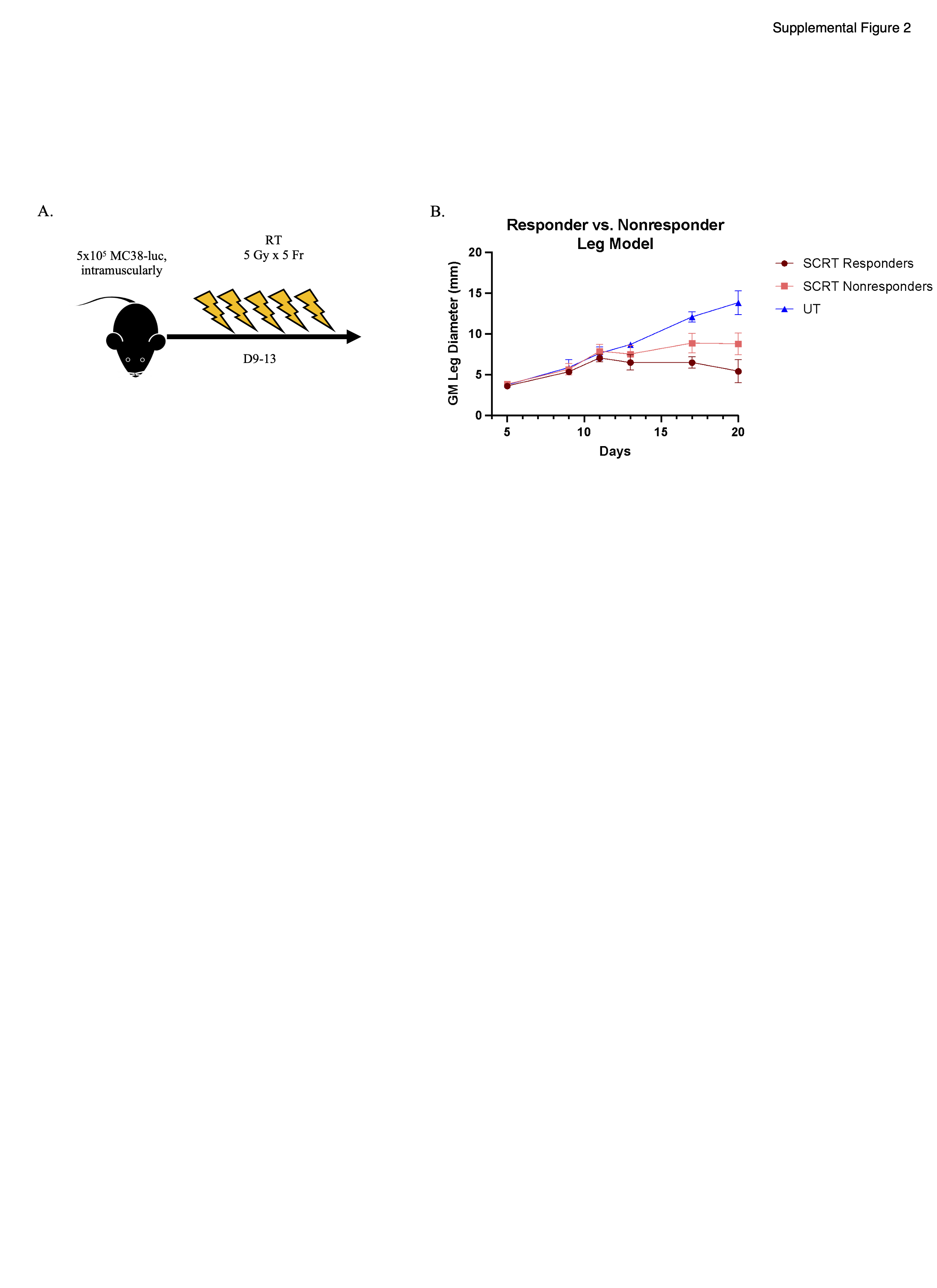

Supplement: Supplementary file 4 — Supplemental Figure 2 [file 41419_2023_5999_MOESM4_ESM.tif]

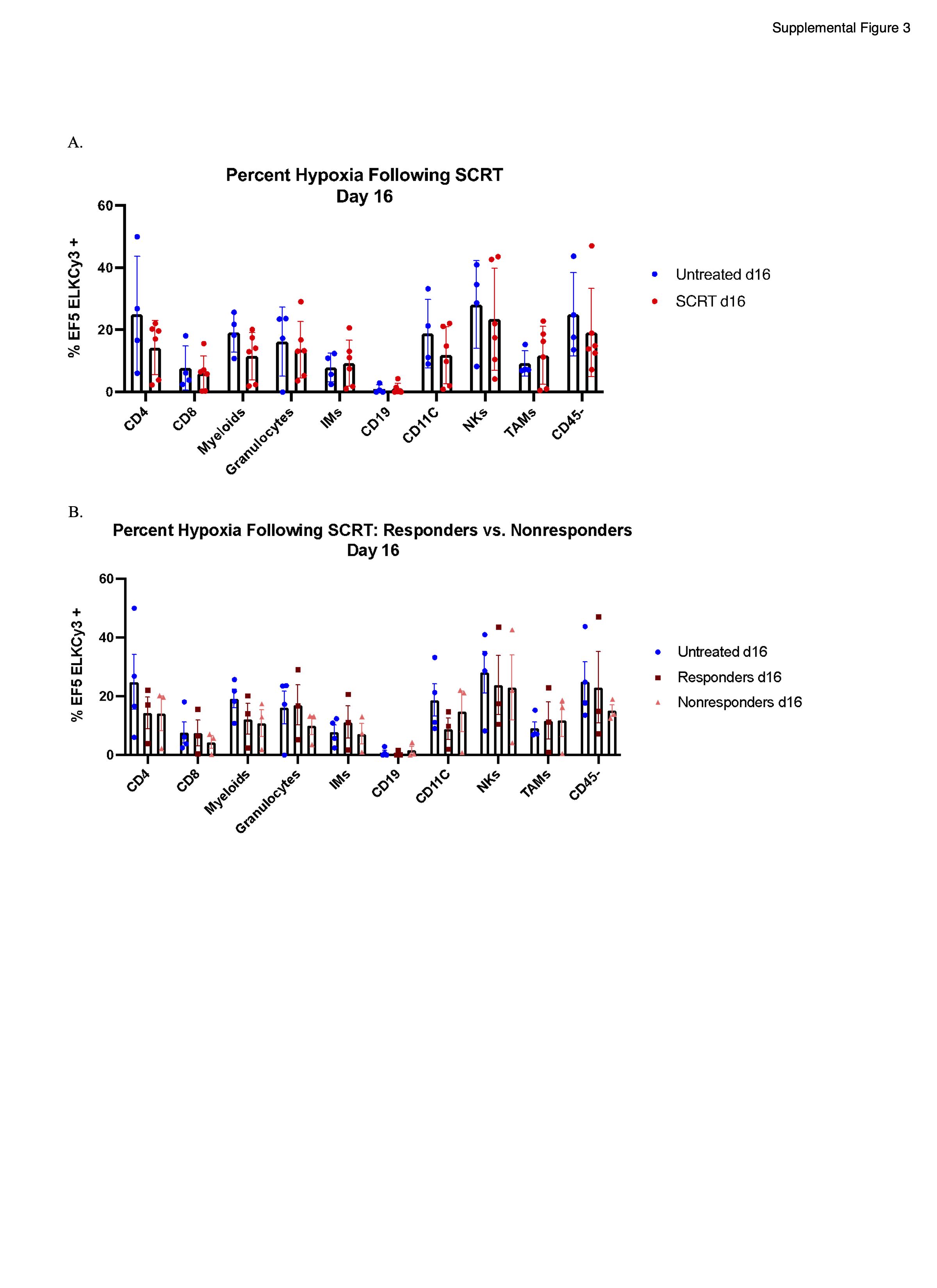

Supplement: Supplementary file 5 — Supplemental Figure 3 [file 41419_2023_5999_MOESM5_ESM.tif]

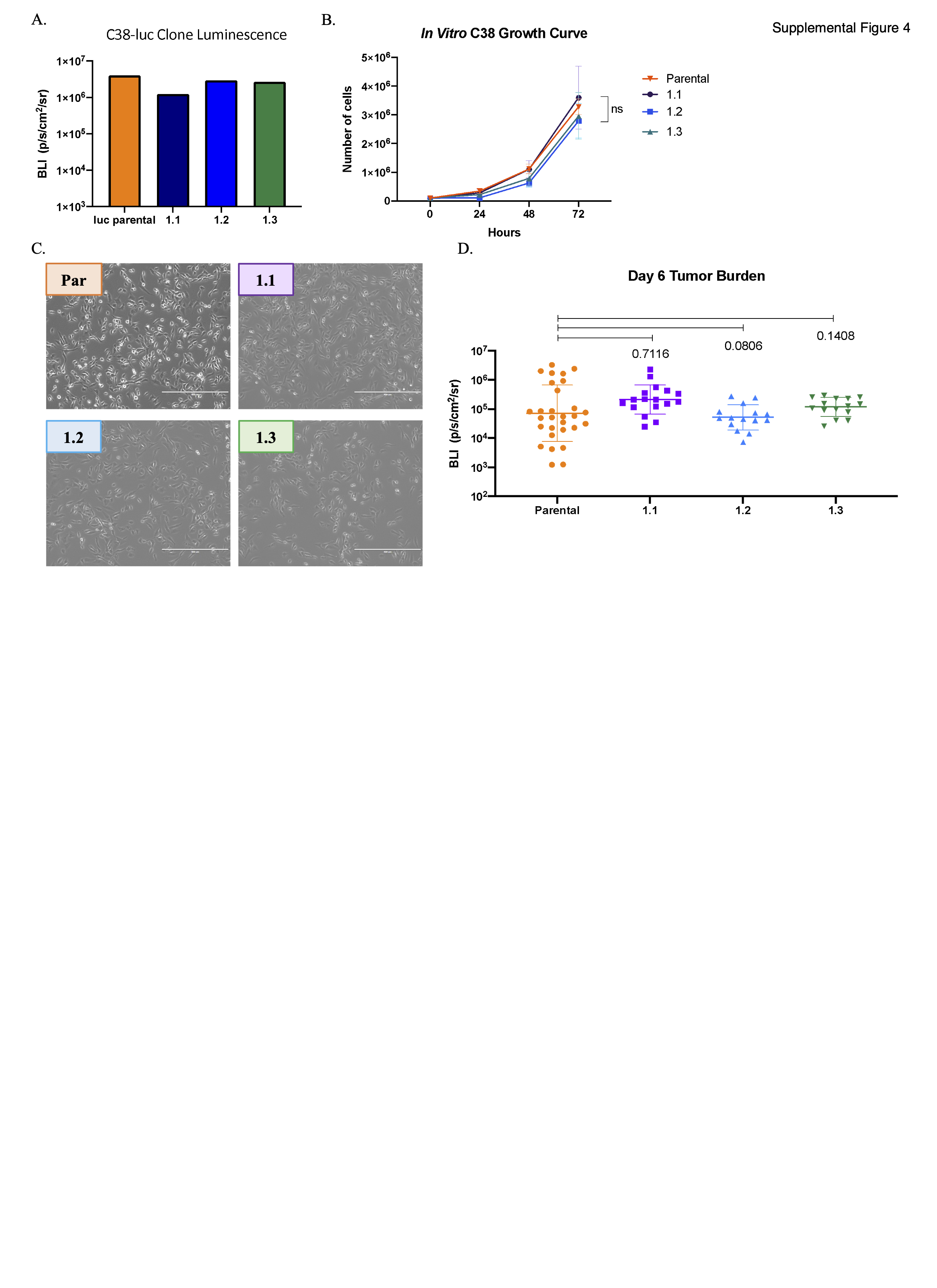

Supplement: Supplementary file 6 — Supplemental Figure 4 [file 41419_2023_5999_MOESM6_ESM.tif]

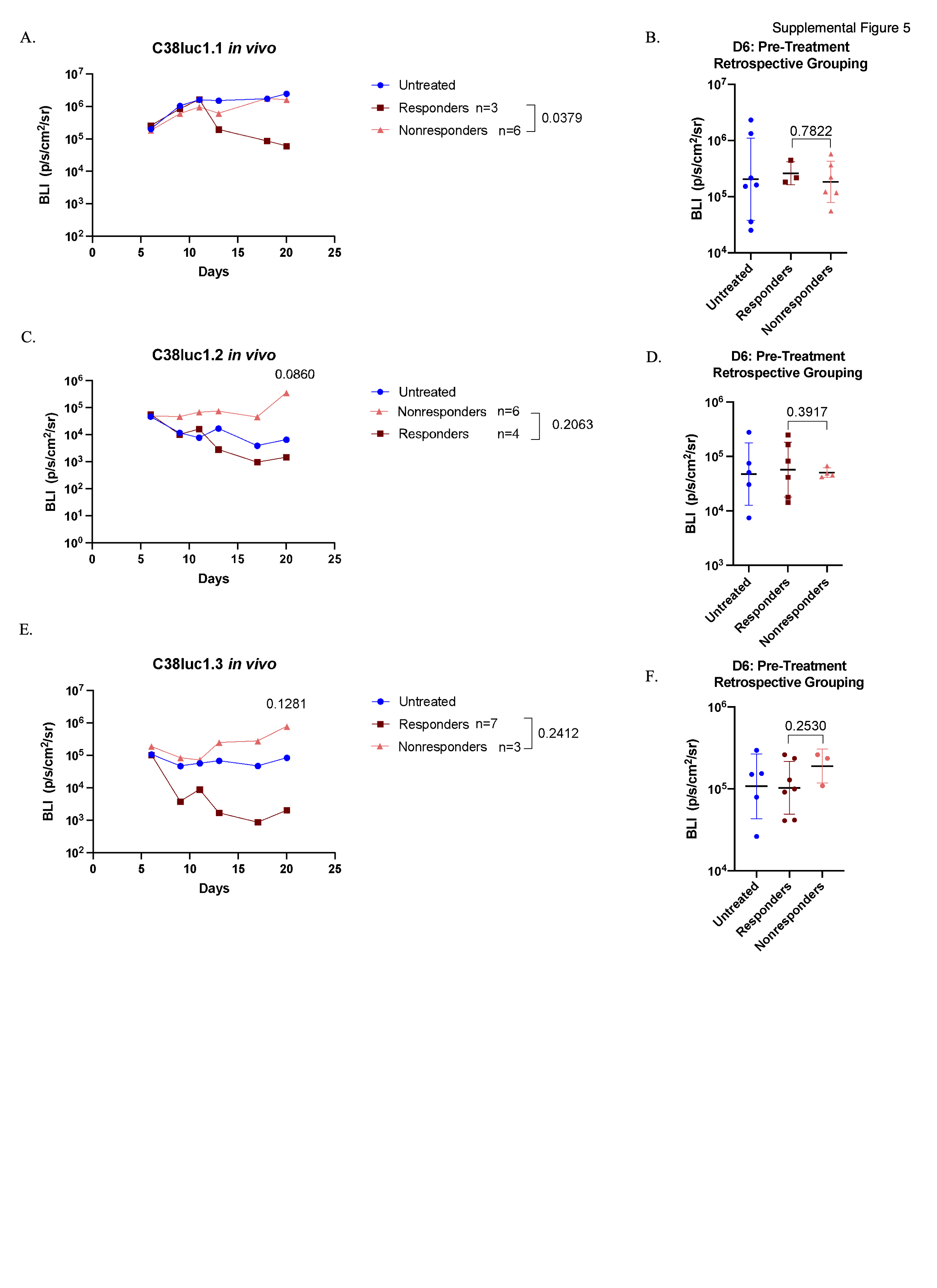

Supplement: Supplementary file 7 — Supplemental Figure 5 [file 41419_2023_5999_MOESM7_ESM.tif]

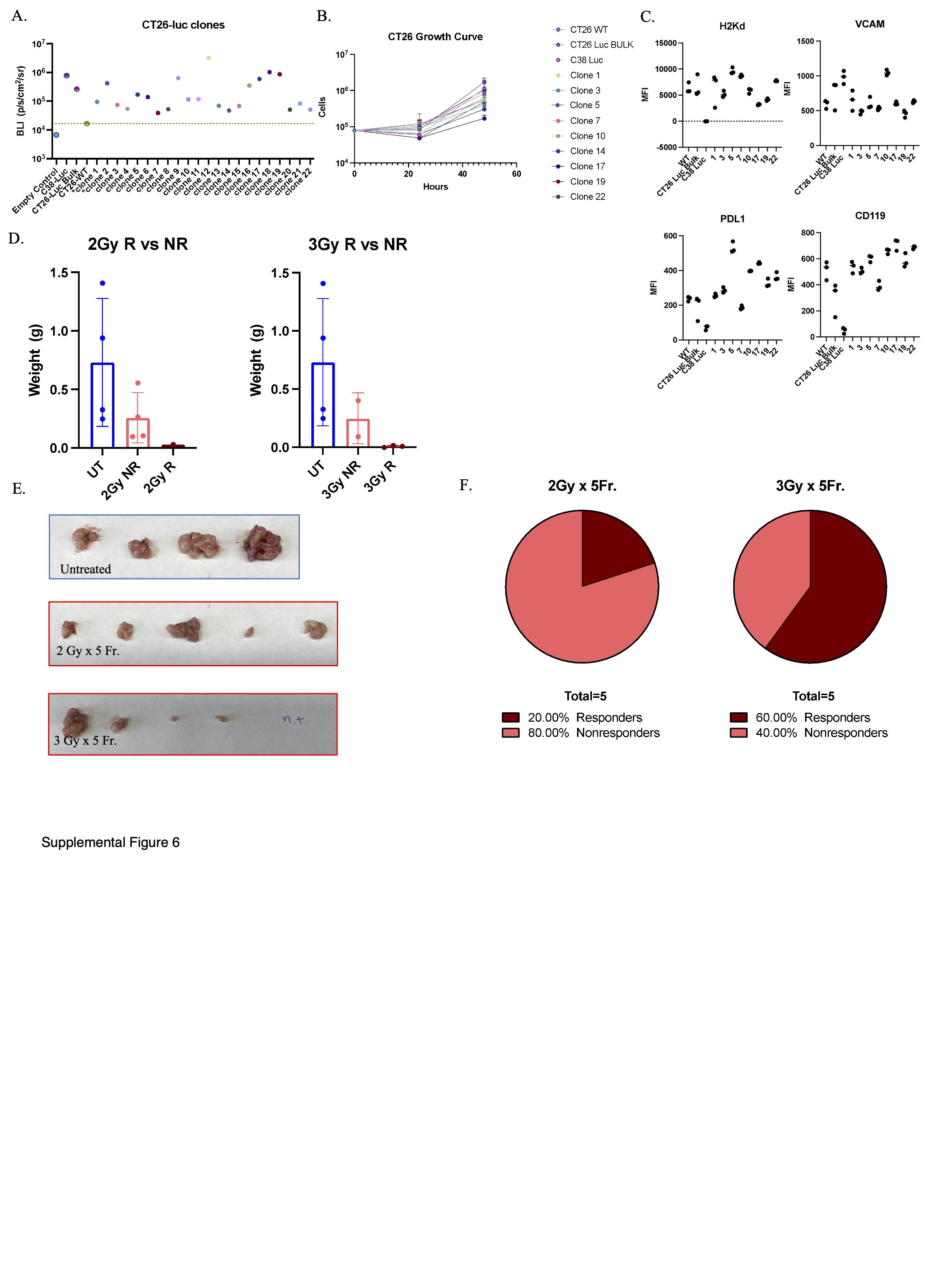

Supplement: Supplementary file 8 — Supplemental Figure 6 [file 41419_2023_5999_MOESM8_ESM.tif]

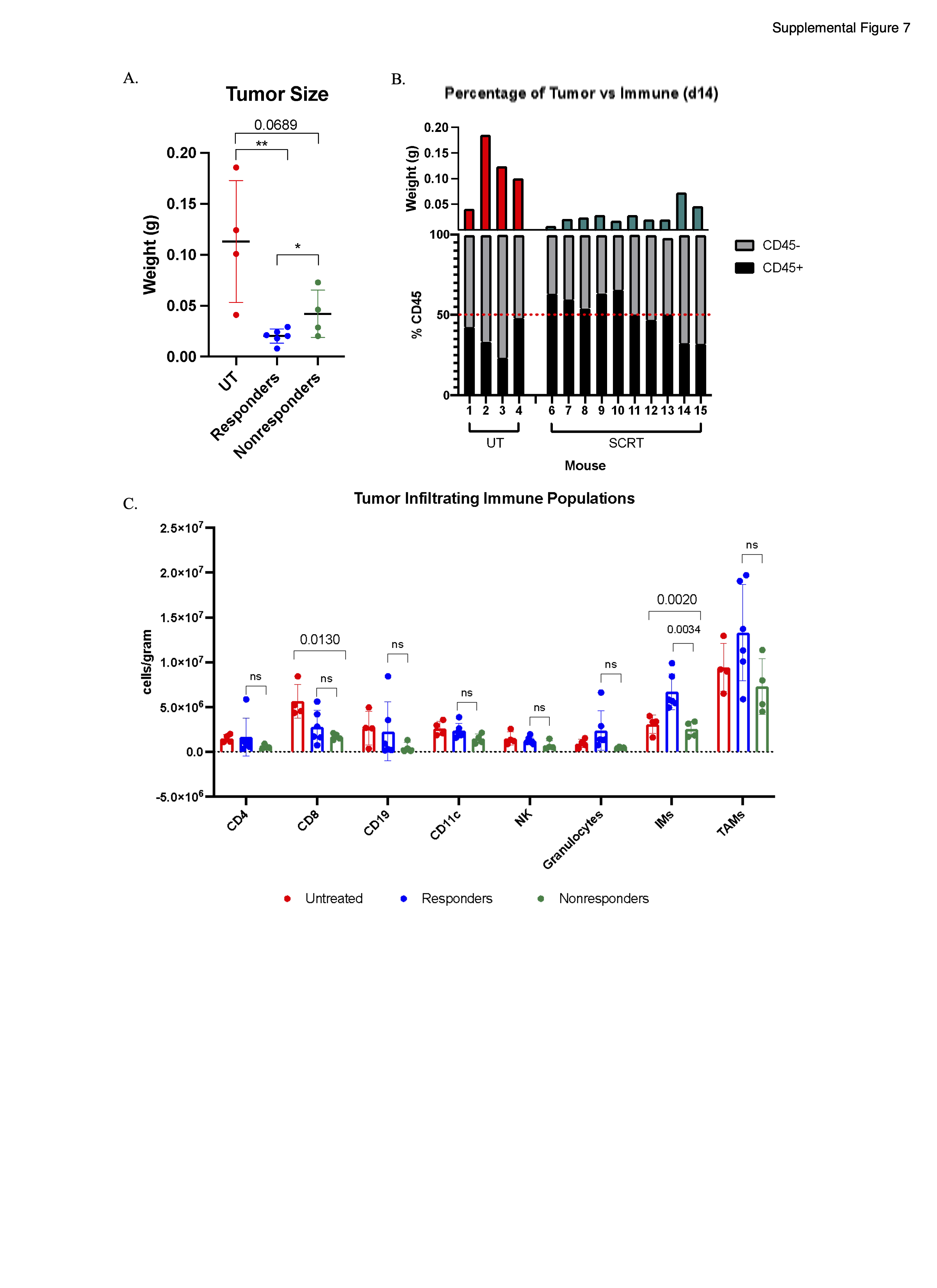

Supplement: Supplementary file 9 — Supplemental Figure 7 [file 41419_2023_5999_MOESM9_ESM.tif]

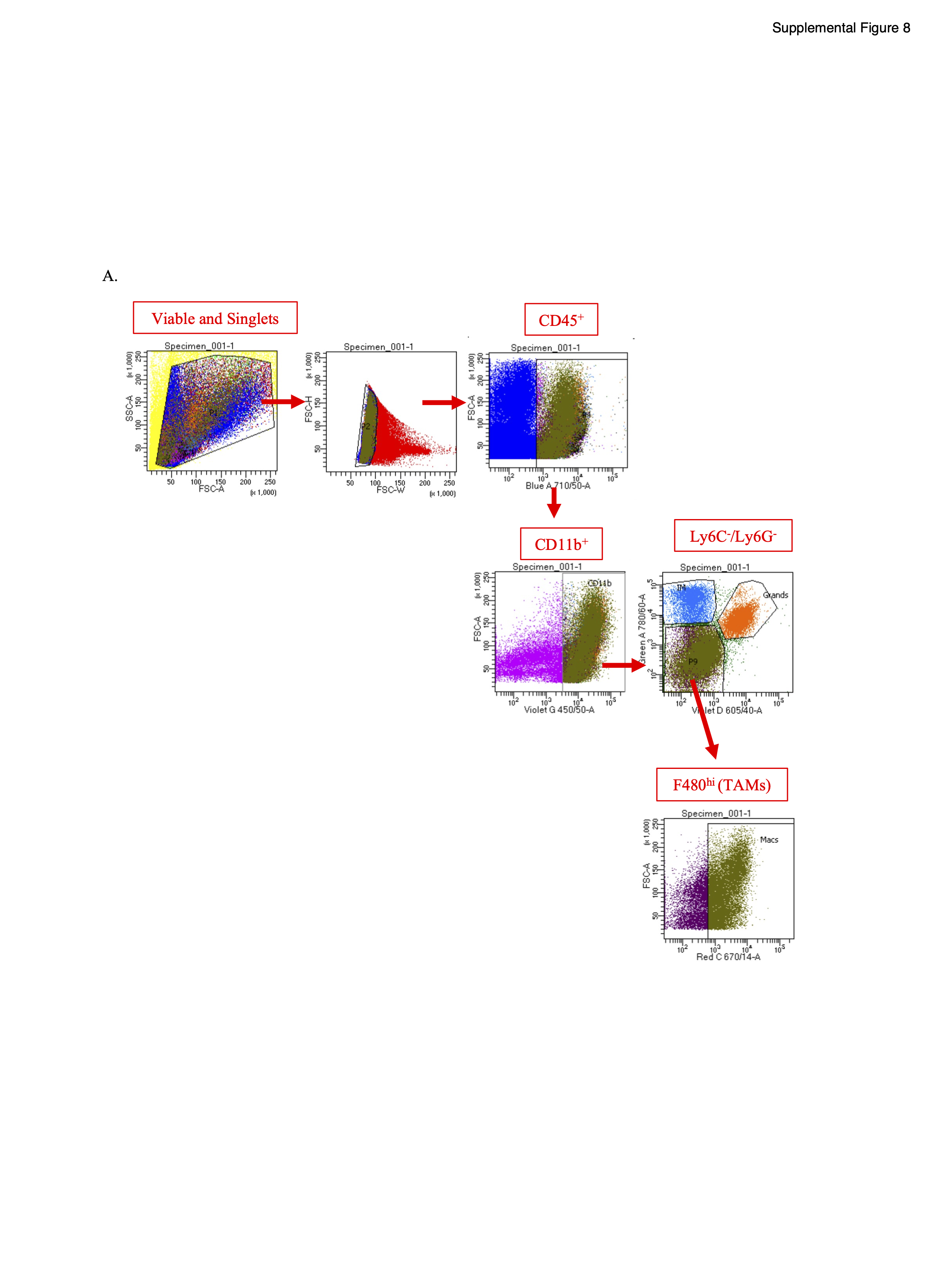

Supplement: Supplementary file 10 — Supplemental Figure 8 [file 41419_2023_5999_MOESM10_ESM.tif]

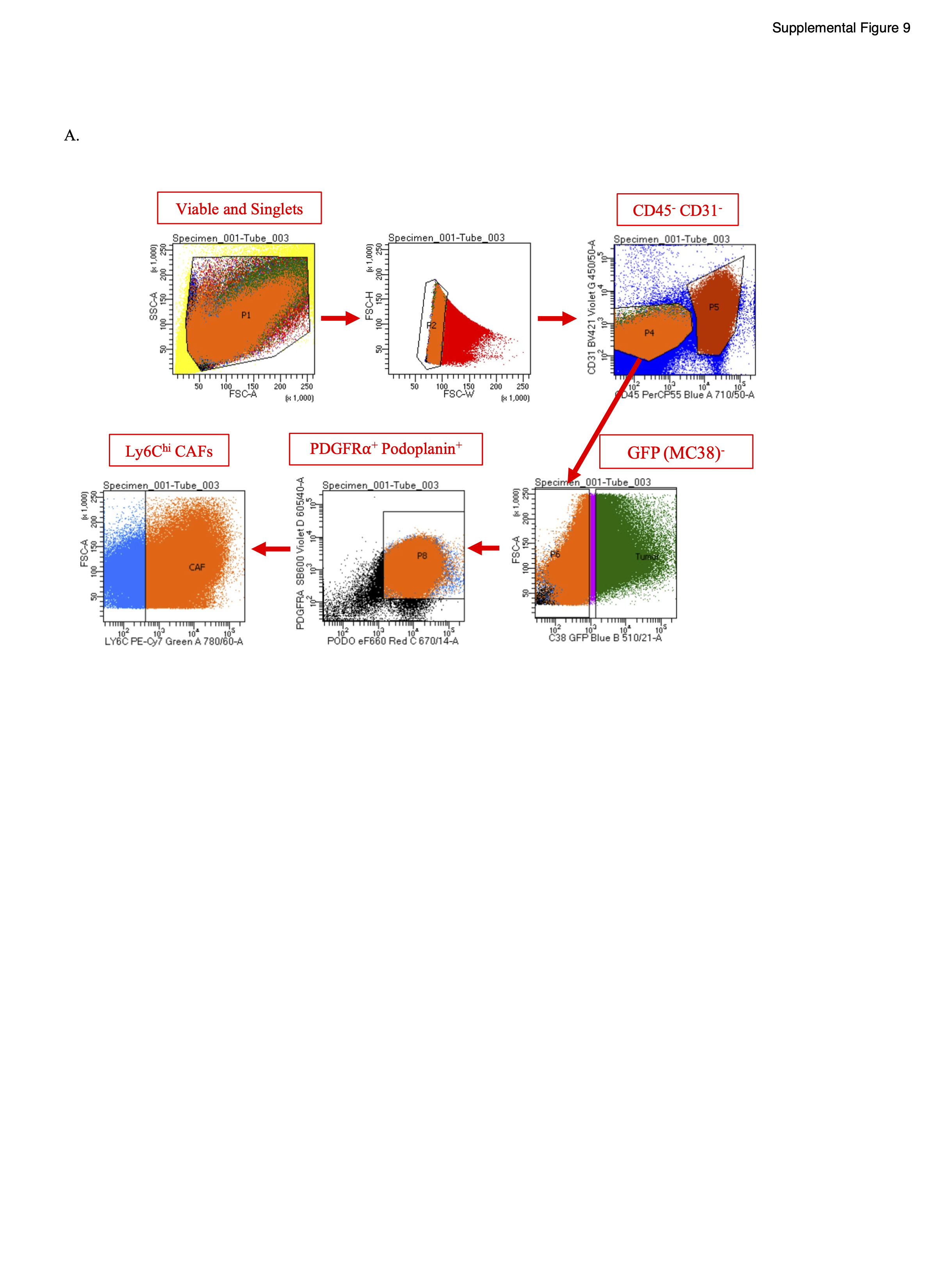

Supplement: Supplementary file 11 — Supplemental Figure 9 [file 41419_2023_5999_MOESM11_ESM.tif]

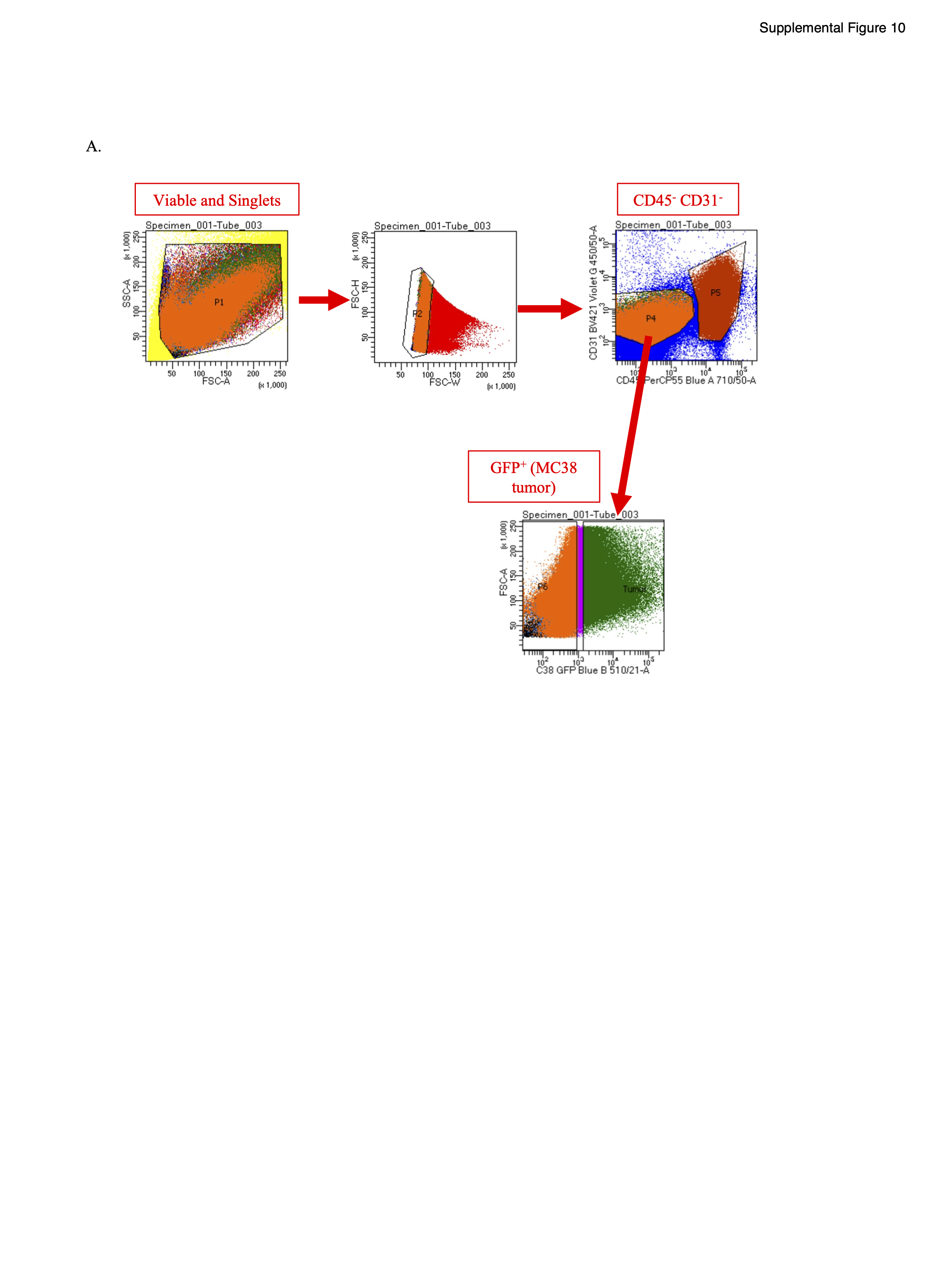

Supplement: Supplementary file 12 — Supplemental Figure 10 [file 41419_2023_5999_MOESM12_ESM.tif]

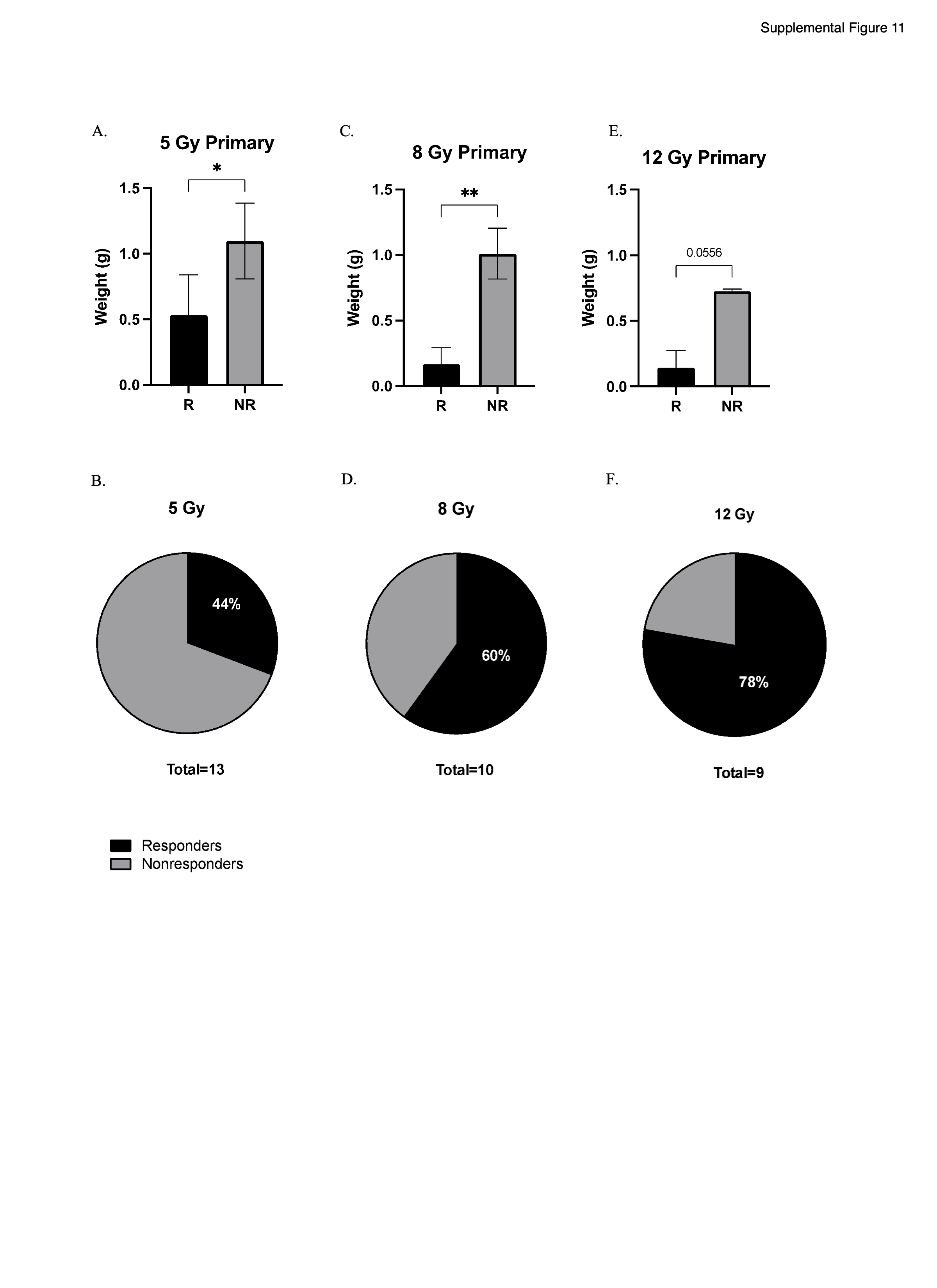

Supplement: Supplementary file 13 — Supplemental Figure 11 [file 41419_2023_5999_MOESM13_ESM.tif]

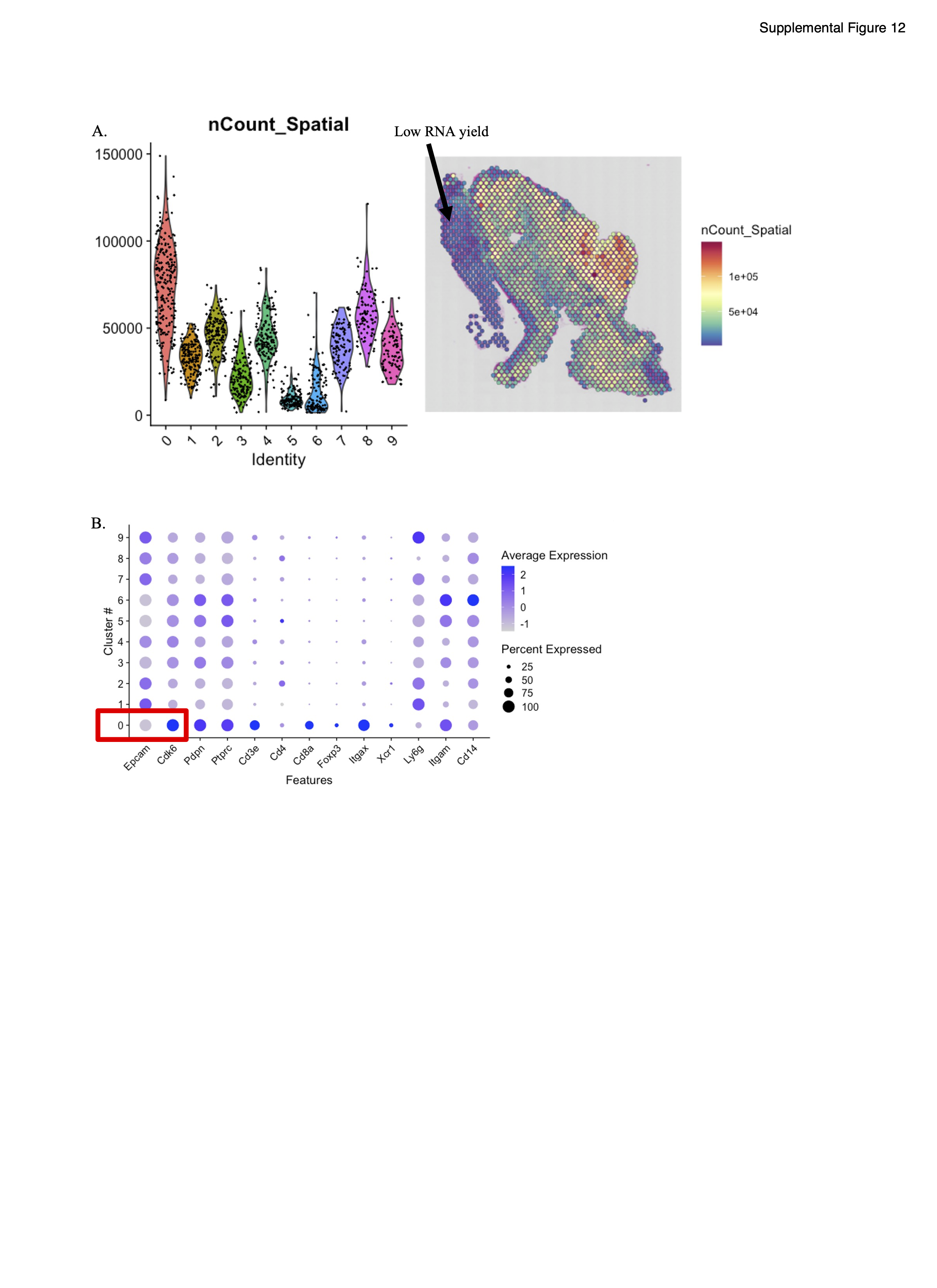

Supplement: Supplementary file 14 — Supplemental Figure 12 [file 41419_2023_5999_MOESM14_ESM.tif]

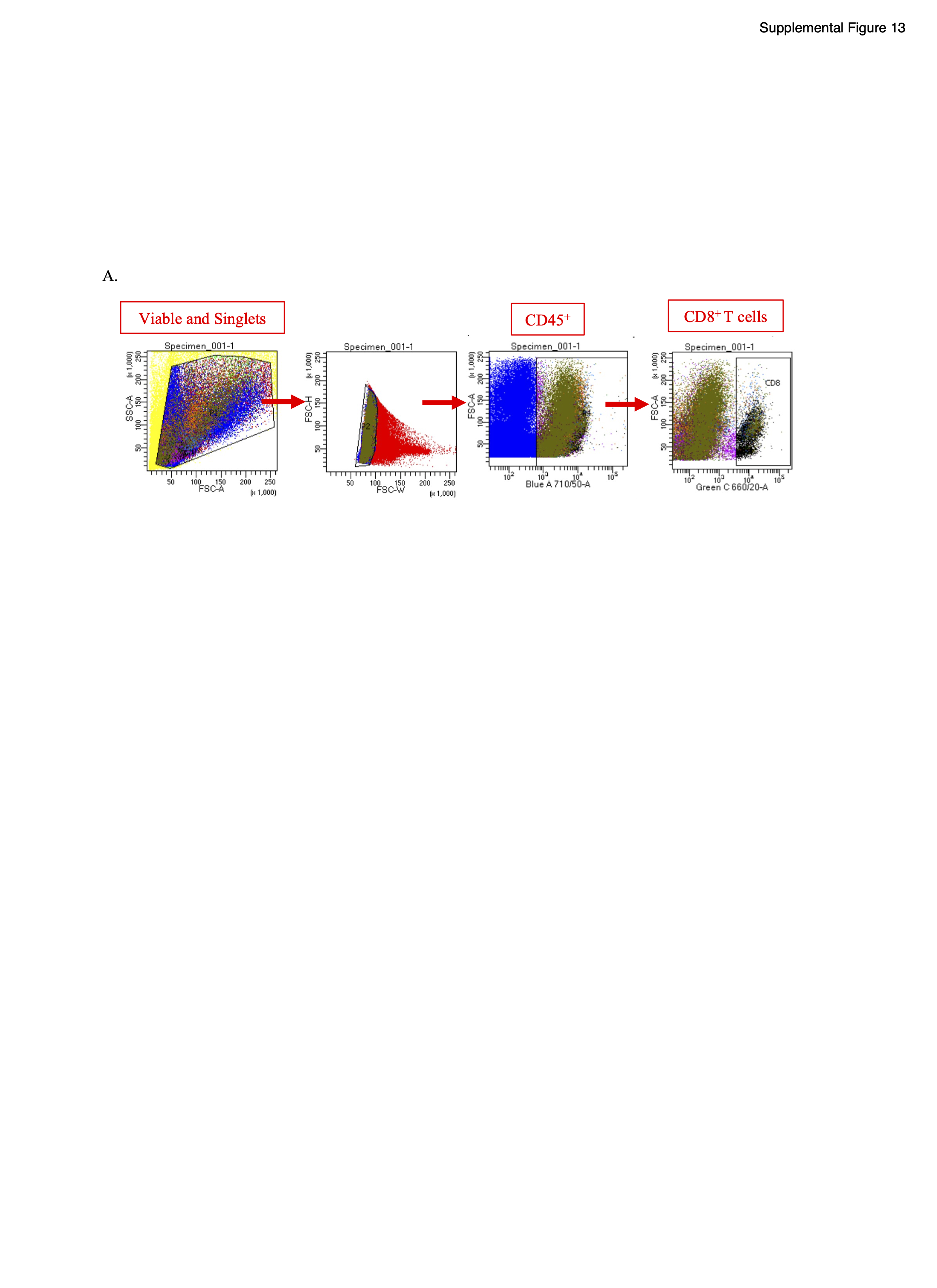

Supplement: Supplementary file 15 — Supplemental Figure 13 [file 41419_2023_5999_MOESM15_ESM.tif]

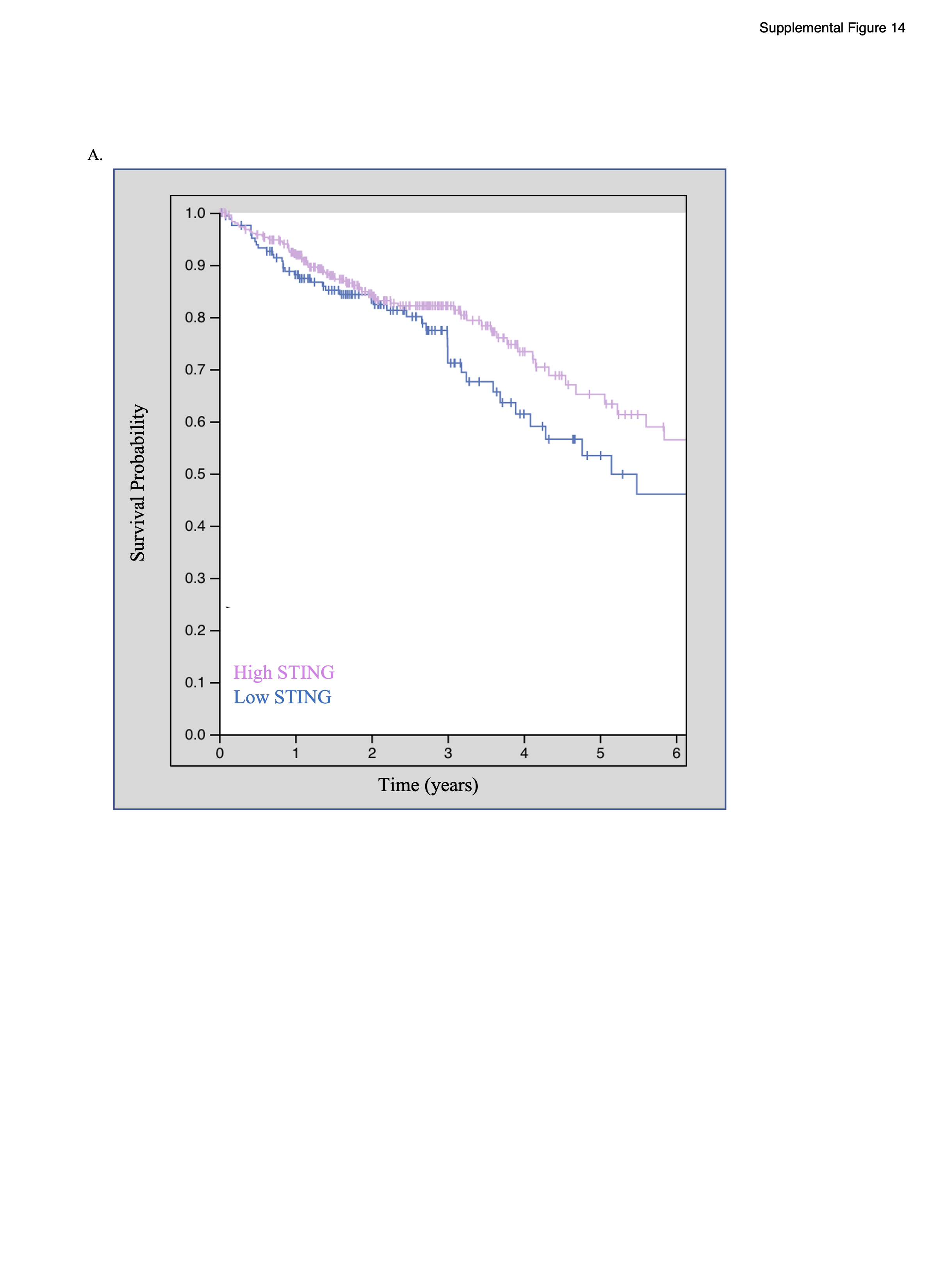

Supplement: Supplementary file 16 — Supplemental Figure 14 [file 41419_2023_5999_MOESM16_ESM.tif]
