# Supplementary material for: New insights into the responder/nonresponder divide in rectal cancer: Damage-induced Type I IFNs dictate treatment efficacy and can be targeted to enhance radiotherapy
Source: Cell Death Dis. 2023 Jul 26;14(7):470. doi: 10.1038/s41419-023-05999-3 (PMC10372053; doi:10.1038/s41419-023-05999-3)
Supplement: Supplementary file 17 — Supplemental Figure Legends [file 41419_2023_5999_MOESM17_ESM.docx]

**Supplemental Figure 1: Phosphorylated ɣH2A.x is upregulated at the site of RT, indicating precise targeting.** (A) Mice injected intrarectally with 2.5x10^4^ MC38-luc cells on day 0. Titanium fiducial clips incorporated on either side of the tumor at day 8, and a single fraction of radiation therapy (RT; 5 Gray) administered on day 9. Three hours following RT, tumors and adjacent rectal tissue harvested and stained by flow cytometry using an anti–pɣH2A.x antibody. (B) Contour plot of pɣH2A.x expression and (C) histograms plotting mean fluorescent intensity (MFI). (D) Quantification of pɣH2A.x MFI plotted for tumor and adjacent rectal tissue.

N = 8 untreated, 9 irradiated

D – Statistical significance determined by ANOVA.

**Supplemental Figure 2: Ectopic model of RC demonstrates responder/nonresponder phenotype. (**A) Intramuscular (i.m.) targeting scheme: 5x10^5^ MC38-luc cells injected i.m. in the left hind leg on day 0 and mice treated with fractionated radiotherapy (RT; 5 Gy/ fraction, 5 consecutive fractions) on days 9-13. (B) Caliper measurements of hind leg diameter. Mice grouped as responders or nonresponders based on tumor burden.

N = 5 untreated and 10 irradiated

**Supplemental Figure 3: There is no differences in the level of intratumoral hypoxia between responders and nonresponders following SCRT.** (A) Tumor bearing mice treated with or without SCRT injected with EF5 (to label regions of hypoxia) and sacrificed on day 16. Tumors harvested and stained for flow cytometry and the percentage of EF5-ELK-Cy3+ cells plotted for each immune population. (B) The percentage of EF5 ELK-Cy3+ cells plotted for each immune population.

N = 4 untreated, 6 irradiated (3 responders, 3 nonresponders).

A, B – Statistical significance determined by Students T test or ANOVA. Not significantly different.

**Supplemental Figure 4: MC38-luc clones exhibit similar characteristics to MC38-luc parental, *in vitro* and *in vivo*.** Three clones selected from the bulk population (MC38-luc parental). (A) Luciferase expression for each clone (1.1, 1.2, 1.3) measured by bioluminescence (BLI), *in vitro*. (B) Growth kinetics of the three clones and MC38-luc parental grown *in vitro* and counted every 24 hours for 3 days; n = 3 (C) Representative images showing morphology of the clones and MC38-luc parental when grown in a 10mm dish. (D) MC38-luc parental and clones 1.1, 1.2, and 1.3 injected orthotopically and tumor burden measured by *in vivo* imaging systems (IVIS) on day 6; n = 29, 17, 15, 15.

B, D – Statistical significance determined by ANOVA.

**Supplemental Figure 5: MC38-luc clones divide into responders and nonresponders following SCRT.** (A, C, E) Clones 1.1, 1.2, or 1.3 injected intrarectally (2.5x10^4^/ mouse) and mice received targeted SCRT from days 9-13. Tumor burden monitored by *in vivo* imaging systems (IVIS). (B, D, F) Animals retrospectively grouped as responders or nonresponders prior to SCRT (day 6).

N = 5 untreated, 10 irradiated

A-F – Statistical significance determined by ANOVA (A, C, E on day 20)

**Supplemental Figure 6: Monoclonal CT26-luc clones selected based on *in vitro* and *in vivo* characteristics and injected *in vivo***. (A) Luciferase expression calculated for 22 clones of luciferase expression CT26 *in vitro* by bioluminescence (BLI). (B) Clones grown *in vitro* for 3 days and counted every 24 hours. (C) 9 clones selected and expression of H2Kd, VCAM-1, PDL-1, and CD119 determined by flow cytometry. (D-F) 1.5 x10^5^ CT26-luc cells injected intrarectally and treated with 5 fractions of either 2 Gy or 3 Gy per fraction from days 9-13. (D) Mice sacrificed on day 19 and primary tumor weight recorded. (E) Images of harvested tumors from day 19. (F) The ratio of responders to nonresponders calculated for each dose.

N = 4 untreated, 5 irradiated

**Supplemental Figure 7: There are no differences in the number of tumor-infiltrating myeloid cells following SCRT.** (A) Untreated (UT) or irradiated (SCRT; d9-13) tumors harvested on day 14 and weighed. (B) The ratio of CD45+ immune cells to CD45- tumor cells plotted for each sample. (C) Immune subsets from untreated or irradiated (SCRT; d9-13) tumors harvested on day 14 and normalized per gram of tumor for untreated, responding, and nonresponding tumors plotted as a bar graph.

N = 4 untreated, 10 irradiated (6 responders, 4 nonresponders)

A, C – Statistical significance determined by ANOVA.

**Supplemental Figure 8: CD11b, Ly6C, Ly6G and F480 identified TAMs.** (A) CD11b+ Ly6C- Ly6G- F480+ tumor associated macrophages (TAMs) sorted from untreated or irradiated (SCRT; d9-13) tumor homogenates on day 14.

**Supplemental Figure 9: PDGFR⍺, podoplanan, and Ly6C identified CAFs.** (A) CD45- CD31- GFP- Podoplanin+ PDGFR⍺+ Ly6C+ cancer associated fibroblasts (CAFs) sorted from *ex vivo* untreated and irradiated (SCRT; d9-13) tumors on day 14.

**Supplemental Figure 10: GFP identified tumor cells.** (A) CD45-, CD31-, GFP+ MC38 tumor cells sorted from *ex vivo* untreated and irradiated tumors (SCRT; d9-13) harvested on day 14.

**Supplemental Figure 11: Increased doses of SCRT result in an increased ratio of responders**. (A, C, E) Tumors harvested on day 27 following fractionated RT from days 9-13 (A – 5 Gy x 5Fr.; C – 8 Gy x 5 Fr.; E – 12 Gy x 5 Fr.). (B, D, F) The ratio of responders to nonresponders plotted in a pie chart.

N = 5 nonresponders, 4 responders for 5 Gy; 4 nonresponders, 6 responders for 8 Gy; 2 nonresponders, 7 responders for 12 Gy.

A, C, E – Statistical significance determined by T test.

**Supplemental Figure 12: Spatial transcriptomics identified 9 distinct clusters in the TME.** (A) RNA yield determined that regions 5 and 6 had low RNA levels, which correlated with areas of necrosis identified by the arrow. (B) A bubble plot was created for each cluster based on expression of key populations within the tumor microenvironment (TME): Epcam as a marker of normal rectal tissue, Cdk6 (cytokeratin 6) as a marker of tumor cells, Pdpn (podoplanin) for cancer associated fibroblasts (CAFs), Ptprc (CD45) as a general immune marker, CD3e/CD4/CD8a/FoxP3 as markers of distinct T cell populations, Itgax (CD11c) as a marker of dendritic cells (DC), Xcr1 as a marker of antigen presenting cells, Ly6g to classify neutrophils, Itgam (CD11b) as a pan myeloid marker, CD14 as a monocyte/macrophage marker.

One individual sample from N = 3

**Supplemental Figure 13 CD8 identified CD8+ T cells.** (A) CD45+, CD8+ T cells sorted from untreated or irradiated (SCRT; d9-13) tumor homogenates on day 14.

**Supplemental Figure 14: High STING1 expression correlates with enhanced survival.** (A) Human colorectal cancer (CRC) survival collected from the Human Protein Atlas and stratified by STING1 expression. Pink curve signifies patients with high STING1 expression while blue curve represents patients with low STING1 expression.
